# A physiology-based mathematical model of the renin-angiotensin system, bone remodeling, and calcium homeostasis: Effects of estrogen and renin-angiotensin system inhibitors

**DOI:** 10.1371/journal.pcbi.1013101

**Published:** 2026-09-22

**Authors:** Melissa M. Stadt, Victoria A. Ralph, Anita T. Layton

**Affiliations:** 1 Department of Applied Mathematics, University of Waterloo, Waterloo, Ontario, Canada; 2 Cheriton School of Computer Science, Department of Biology, School of Pharmacology, University of Waterloo, Waterloo, Ontario, Canada; University at Buffalo - The State University of New York, UNITED STATES OF AMERICA

## Abstract

During menopause, estrogen levels decline significantly, leading to substantial physiological changes due to estrogen’s regulatory role in various systems. In particular, estrogen helps prevent excessive bone resorption by its impact on bone remodeling. When estrogen levels decrease, bone resorption increases, often resulting in weakened bones and osteoporosis in post-menopausal women. Experimental studies have also shown that estrogen regulates the renin-angiotensin system (RAS), a hormone system involved in many physiological processes, including blood pressure regulation. Additionally, the RAS has an interconnected relationship with calcium regulatory and bone remodeling systems. Given these dynamic interplays, we developed a physiology-based mathematical model that simulates the interactions of estrogen, key RAS components, calcium regulation, and bone remodeling to investigate how perturbations in one system affect the others. This represents the first known model to include the direct impacts of estrogen on the RAS and to couple the RAS with calcium homeostasis and bone remodelling systems in humans. Through model simulations and sensitivity analysis, we investigated how declining estrogen levels affect the RAS and bone mineral density. Furthermore, we quantified how RAS inhibitors, specifically angiotensin-converting enzyme inhibitors (ACEi) and angiotensin receptor blockers (ARB), may increase bone density during post-menopausal estrogen decline. Our simulation results suggest that ARB treatment may be more effective at improving bone mineral density during postmenopausal estrogen decline, and that this trend remains consistent regardless of the age of RAS inhibitor initiation.

## Author summary

Post-menopausal estrogen decline is associated with reduced bone mineral density and increased risk of osteoporosis. In addition to its established role in bone metabolism, estrogen also regulates the renin-angiotensin system (RAS), a hormonal system primarily involved in blood pressure regulation. Experimental studies suggest that interactions between these physiological systems contribute to changes in bone remodeling during menopause. To investigate these dynamic interactions, we developed a physiology-based mathematical model that integrates the RAS with calcium homeostasis and bone remodeling in humans. We examined how reductions in estrogen affect bone remodeling pathways and influence bone mineral density. We also evaluated the potential effects of commonly prescribed RAS inhibitors on bone health. Our simulations predict that estrogen decline increases signaling pathways associated with bone resorption and accelerates bone loss. However, RAS inhibitors can reduce these effects and mitigate menopause related bone mineral density loss. The results further suggests that angiotensin receptor blockers may provide greater protection against bone mineral density loss than angiotensin-converting enzyme inhibitors. These findings support emerging clinical evidence linking the RAS to skeletal health and could inform the prescription of RAS inhibitors for postmenopausal women.

## Introduction

Bone remodeling is a complex physiological process that maintains the balance between bone formation and resorption. This process is mediated by bone cells: osteoblasts, which are responsible for bone formation, and osteoclasts, which are responsible for bone resorption. The activity and proliferation of osteoblasts and osteoclasts is regulated by a system of various receptors, their corresponding ligands, and hormonal signals [1,2]. One such regulatory system is the RANK-RANKL-OPG system. Receptor activator of nuclear factor kappa-B ligand (RANKL) is expressed by osteoblasts and binds to receptor activator of nuclear factor kappa-B (RANK) on osteoclasts to promote osteoclast differentiation and activation. Osteoprotegerin (OPG) is a decoy receptor that binds to RANKL, preventing its binding with RANK and thereby inhibiting osteoclast activity. The balance between RANKL and OPG is essential for maintaining bone homeostasis as an increase in RANKL or OPG can lead to excessive or reduced bone resorption, respectively [1].

Estrogen impacts bone remodeling by inhibiting osteoclast activity, thereby protecting bones from excessive resorption. When estrogen levels in women decline post-menopause, osteoclast activity increases, resulting in increased resorption, and ultimately weakened bones. This often leads to post-menopausal osteoporosis. Post-menopausal osteoporosis is the most common form of osteoporosis, which is characterized by increased bone fragility, making fractures more likely from minor trauma or in severe cases, even spontaneous fracture. It is estimated that about 1 in 3 women over 50 years of age will suffer a fracture related to post-menopausal osteoporosis [3,4].

The main component of bone structure is hydroxyapatite, a compound made up of phosphate and calcium. Given their role in developing the key structure of bones, maintaining balanced levels of calcium and phosphate is critical for bone health. The regulation of calcium and phosphate levels is driven by calciotropic hormones: calcitriol and parathyroid hormone (PTH) [5]. Researchers have reported a bidirectional relationship between the calciotropic hormones and the renin-angiotensin system (RAS) [6], a hormone system that plays a key role in many physiological processes, including blood pressure regulation, in addition to a direct impact of the RAS on bone remodeling [7]. The RAS is a hormone cascade that produces the bioactive peptide angiotensin II (Ang II). Ang II binds to angiotensin type 1 receptors (AT1R) and angiotensin type 2 receptors (AT2R) to trigger various physiological effects. The RAS is also modulated by estrogen levels, and therefore is impacted by the rapidly declining estrogen levels in post-menopausal women [8]. This dysregulation contributes to the higher susceptibility of post-menopausal women to the development of hypertension, cardiovascular disease, and osteoporosis [8].

Inhibition of RAS components, particularly angiotensin-converting enzyme (ACE) and AT1R binding, has shown to be effective targets for antihypertensive treatment [9]. These drugs are classified as RAS inhibitors. Experimental data has shown that the use of RAS inhibitors improves bone quality independent of their effect on blood pressure [10,11]. Given that RAS inhibitors are relatively well tolerated, safe, and are reasonably priced drugs, there has been interest in using RAS inhibitors to prevent osteoporosis in post-menopausal women [10–12]. Additionally, since hypertension is highly prevalent in post-menopausal women, understanding the impacts of antihypertensive treatments, such as RAS inhibitors, on bone remodeling is important for achieving blood pressure control and bone health in post-menopausal women [10–12].

Many mathematical models have been developed to study bone remodeling as recently reviewed in [2]. Peterson and Riggs [13] pioneered a mathematical model of calcium homeostasis coupled to a detailed bone biology model. Their model consists of bone, renal, and blood compartments to predict levels of PTH, calcitriol, calcium, phosphate, and bone remodeling activity. This model has been applied to investigate applications related to bone remodeling and calcium homeostasis, including the impact of estrogen [14], mineral bone disorder in patients with chronic kidney disease [15], and the impact of fibroblast growth factor 23 (FGF-23) and vascular calcification [16]. Mathematical models have also been developed for the RAS to understand its role in blood pressure regulation. Specifically, Lo et al. [17] developed a model of the RAS in humans to investigate the differences between normotensive and hypertensive patients. This model was later included in a full body blood pressure model by Hallow et al. [18]. Leete et al. [19] further developed this model to investigate the effects of sex-based differences in the RAS on blood pressure regulation. However, no known models have been developed that include the direct impact of estrogen on the RAS or have coupled the RAS impacts with calcium homeostasis and bone remodeling in humans.

In this study, we (i) investigate the impact of age-related estrogen decline by including the impact of estrogen on the RAS and (ii) couple the calcium homeostasis and bone remodeling model [13,14] with the RAS model [17–19] to predict the impact of RAS inhibitors on this system.

## Materials and methods

In this study, we develop a coupled ordinary differential equation model that represents (i) the impact of estrogen on the RAS (Section Impact of estrogen decline in aging women on the RAS) and (ii) couple this estrogen-RAS model with a bone biology and calcium homeostasis model (Section Composite calcium homeostasis, bone remodeling, and RAS model). We then use this model to simulate the effects of post-menopausal estrogen decline on the RAS, bone remodeling, and calcium homeostasis. Additionally, we simulate the impact of RAS inhibitors. To quantify the impact of parameter variation on model output, we conduct a global sensitivity analysis. A model schematic is shown in Fig 1. A list of the model equations and key parameter descriptions are given in S1 Appendix and S1 Table, respectively. The model incorporates multiple organ systems and physiological spaces, including the vasculature, intracellular space, kidneys, lungs, parathyroid gland and bone. Key species in the vasculature include calcium (Ca^2+^), phosphate (PO_4_), calcitriol, PTH, angiotensinogen (AGT), angiotensin I (Ang I), Ang II, and renin. The intracellular space includes PO_4_, while the kidneys contain 1α-hydroxylase (AOH) and the lungs express ACE. The parathyroid gland contains the parathyroid gland PTH pool. The bone system includes osteoclasts, osteoblasts, transforming growth factor-β (TGF-β), RANK, RANKL, and OPG.

**Fig 1:**
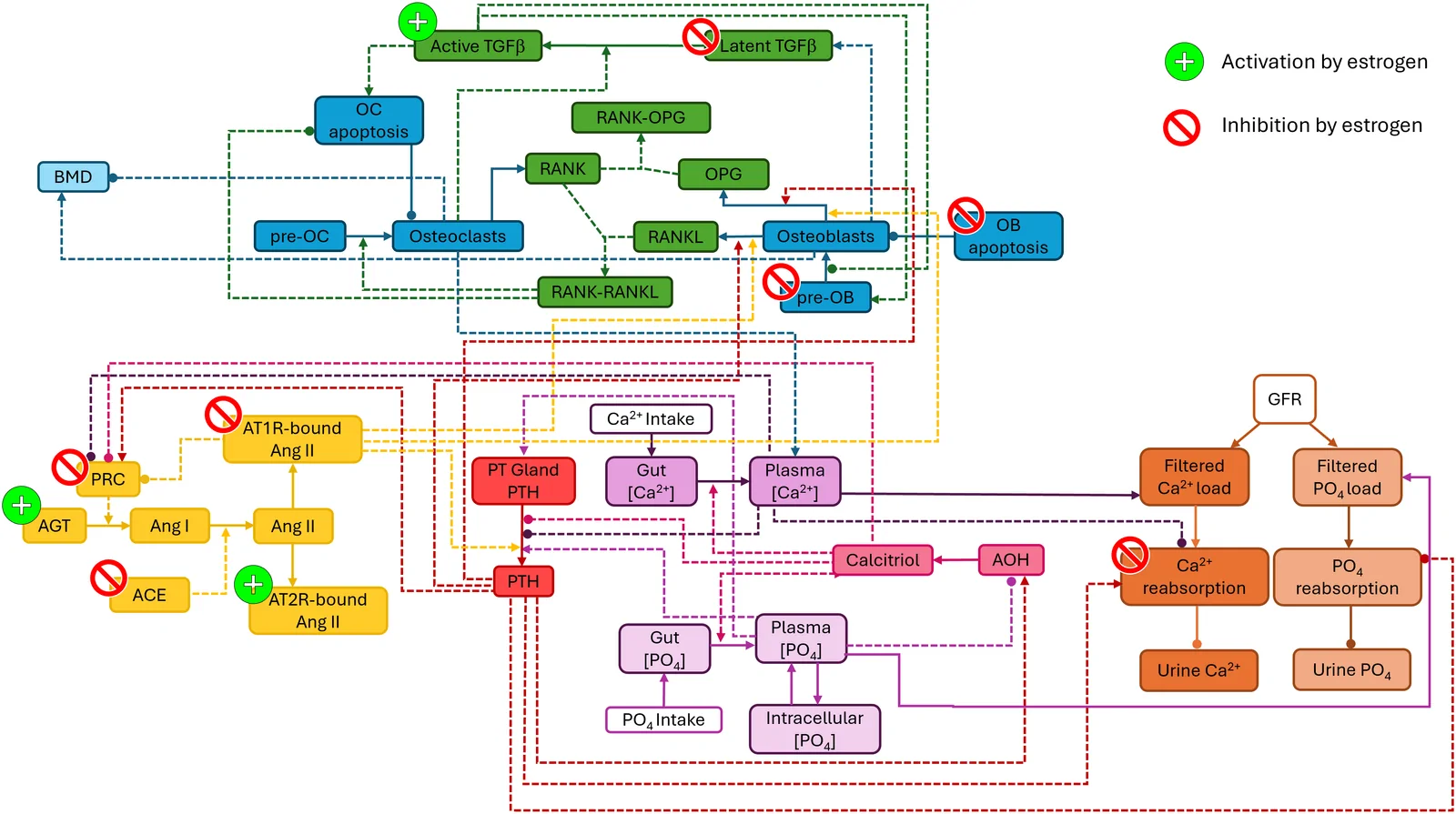
Schematic model of calcium regulation, bone remodeling, the renin-angiotensin system (RAS), and the impact of estrogen on these systems. Purple nodes represent phosphate and calcium levels, orange nodes represent phosphate and calcium renal handling, yellow nodes represent the RAS, red nodes represent parathyroid hormone, pink nodes represent calcitriol, blue nodes represent bone cells and bone mineral density, green nodes represent signaling mechanisms in the bone. Nodes with a white background represent fixed values in the model. The impacts of estrogen are indicated by the red and green symbols for inhibition and activation, respectively. The solid arrows represent flux, secretion, synthesis, or expression, while the dashed lines represent impacts on other biological components. The pointed arrows represent activation and the rounded arrows represent inhibition. ACE: angiotensin-converting enzyme, AGT: angiotensinogen, Ang: angiotensin, AOH: 1α-hydroxylase, AT1R: angiotensin type 1 receptor, AT2R: angiotensin type 2 receptor, BMD: bone mineral density, Ca^2+^: calcium, GFR: glomerular filtration rate, OB: osteoblast, OC: osteoclast, OPG: osteoprotegerin, PO_4_: phosphate, PRC: plasma renin concentration, PT: parathyroid, PTH: parathyroid hormone, RANK: Receptor Activator of Nuclear Factor Kappa-B, RANKL: RANK ligand, TGFβ: transforming growth factor β.

### Renin-angiotensin system model

In this study, we use the RAS model with the female-specific parameter set from [19]. The model variables consist of the systemic concentrations of AGT, Ang I, Ang IV, Ang (1–7), AT1R-bound Ang II, and AT2R-bound Ang II. AT1R-bound Ang II inhibits renin secretion through a feedback loop. For a detailed description of the underlying RAS model used in this study, see [18,19].

#### Impact of estrogen decline in aging women on the RAS.

Estrogen levels impact the RAS by activating and inhibiting various key processes. Specifically, estrogen activates AGT production and AT2R binding, while inhibiting renin secretion, ACE activity, and AT1R binding [8]. These processes are illustrated in Fig 1.

After the onset of menopause, estrogen levels decrease dramatically. To model this decline in estrogen levels, we use the estrogen model for post-menopausal women from [20]. The normalized level of estrogen, denoted by *E*, is modeled through explicit time dependence so that


$$\begin{array}{c} E(t)=\left\{ \begin{array}{cc} 1 & t<t_{e}\\ \frac{1}{1+(t-t_{e})/\tau_{e}} & t\geq t_{e} \end{array}\right. \end{array}$$ (1)

where *t* is the time in years (i.e., age), $t_{e}=50$ years is the age of onset of estrogen decline, and $\tau_{e}=2.6$ years is a parameter fit to normalized estrogen data from [21] that determines the slope of estrogen decline in relation to women’s final menstrual period (see [20] for details). Note that before $t_{e}$, *E* = 1 so that estrogen is assumed to remain at baseline level pre-menopause. Indeed, estrogen levels vary during the menstrual cycle [22]. In this study, we do not consider variations in estrogen over the menstrual cycle in pre-menopausal women and consider baseline estrogen level pre-menopause as the average level through the full cycle.

To include the impact of estrogen on the RAS, we use functions of the form:


$$\begin{array}{c} g_{i}^{+}(E)=V_{max,i}^{+}\cdot \frac{E}{e_{i}+E} \end{array}$$ (2)

$$\begin{array}{c} g_{i}^{-}(E)=V_{max,i}^{-}\cdot \frac{e_{i}}{e_{i}+E} \end{array}$$ (3)

to model activation (*g*^+^) or inhibition (*g*^–^) with


$$\begin{array}{c} V_{max,i}^{+}=e_{i}+1,\;V_{max,i}^{-}=\frac{e_{i}+1}{e_{i}} \end{array}$$

where *i* denotes the target mechanism, $e_{i}$ is a parameter that is determined for each relevant target mechanism, and *E* is given by Eq. 1. These are Hill-type activation and inhibition functions, normalized such that when *E* = 1, $g_{i}^{+}(E)=g_{i}^{-}(E)=1$ for any value of $e_{i}$. Thus, at baseline estrogen levels, there are no changes.

The enzyme renin is secreted by the juxtaglomerular cells of the kidneys to convert AGT to Ang I. Estrogen inhibits renin levels [8,23,24]. We let $g_{renin}^{-}$ be the impact of estrogen on renin secretion. The parameter value for $e_{renin}$ was chosen based on experimental results from [24] which compared plasma estrogen to plasma renin levels under baseline conditions and estrogen injection. Renin secretion is modeled by


$$\begin{array}{c} R_{sec}=N_{rs}\cdot \nu_{AT1R}\cdot g_{renin}^{-}(E) \end{array}$$ (4)

where $N_{rs}$ is baseline renin secretion and $\nu_{AT1R}$ is the feedback of [AT1R-bound Ang II] on renin secretion from [18]. Plasma renin concentration (PRC) is modeled by


$$\begin{array}{c} \frac{d[PRC]}{dt}=R_{sec}-\frac{\ln(2)}{h_{renin}}\cdot [PRC] \end{array}$$ (5)

where $h_{renin}$ denotes the half life of renin as in [18].

AGT is synthesized in the liver and is stimulated by estrogen [8,23,25]. In post-menopausal women, AGT levels decline from pre-menopause levels [23]. To model the impact of estrogen on AGT, we multiply the production of AGT, denoted by $k_{AGT}$, by $g_{AGT}^{+}(E)$. The value of $e_{AGT}$ was based on rat experimental data from [25] which quantified the increase in hepatic AGT mRNA levels under estrogen injection [25]. AGT concentration is modeled by


$$\begin{array}{c} \frac{d[AGT]}{dt}=g_{AGT}^{+}(E)\cdot k_{AGT}-PRA-\frac{\ln(2)}{h_{AGT}}\cdot [AGT] \end{array}$$ (6)

where


$$\begin{array}{c} PRA=X_{PRCPRA}\cdot [PRC]\cdot \frac{\left[AGT\right]}{K_{AGT0}+[AGT]} \end{array}$$ (7)

is plasma renin activity (PRA) with parameter $X_{PRCPRA}$ and $h_{AGT}$ is the half-life for AGT as in [18]. The equation for PRA is a slight modification from the equation used in [18,19] to include the impact of AGT on PRA as well. The $K_{AGT0}$ parameter was fitted to predict the same model output in the original blood pressure model from [19].

ACE converts Ang I into Ang II. Researchers have reported that both ACE expression and activity are inhibited by estrogen in rodent models [26–29]. We let $g_{ACE}^{-}$ represent the inhibition of estrogen levels on ACE activity. The $e_{ACE}$ parameter is based on differences in ACE activity under estrogen injection in ovariectomized rats, as reported in [27]. Ang I can be converted independently of ACE into Ang II by chymases (chym) [30] or into Ang (1–7) by neprilysin (nep) [31]. Ang II can be converted to Ang (1–7) by angiotensin-converting enzyme 2 (ACE2), or into Ang IV by aminopeptidases (II, IV) [32]. The concentration of Ang I and Ang II are modeled by


$$\begin{array}{c} \frac{d[AngI]}{dt}=PRA-(g_{ACE}^{-}(E)\cdot c_{ACE}+c_{chym}+c_{nep})[AngI]-\frac{\ln(2)}{h_{AngI}}[AngI] \end{array}$$ (8)

$$\begin{array}{cc} \frac{d[AngII]}{dt} & =(g_{ACE}^{-}(E)\cdot c_{ACE}+c_{chym})[AngI]\\ & -(c_{ACE2}+c_{II,IV}+g_{AT1}^{-}(E)\cdot c_{AT1}+g_{AT2}^{+}(E)\cdot c_{AT2})[AngII]\\ & -\frac{\ln(2)}{h_{AngII}}[AngII]+k_{inf} \end{array}$$ (9)

where $c_{i}$ denotes the corresponding enzyme activities and $h_{AngI}$ and $h_{AngII}$ are the half lives of Ang I and Ang II, respectively, as in [18]. $k_{inf}$ is an input used for Ang II infusion (see Section Parameter fitting to PTH and Ang II infusion experiments) and is set to 0 in simulations with no Ang II infusion.

Ang II is reported to remain around the same in aging humans [33]. Ovariectomized rats showed a small decrease in plasma Ang II levels, which was reversed by estrogen replacement at pre-ovariectomy levels [34,35]. We note that Ang II levels are often reported in post-menopausal women on estrogen replacement therapy, but researchers have found significant differences in the impact of estrogen replacement therapy on the RAS when taking via oral or transdermal therapy [36]. This is likely due to the high exposure to estrogen in the liver when ingesting oral estrogen.

Ang II binds to AT1R and AT2R, triggering different physiological responses. Estrogen inhibits Ang II binding to AT1R, but activates Ang II binding to AT2R [8,29,37]. AT1R density is increased over 2-fold in ovariectomized rats [38]. Conversely, AT2R receptor density measured in the left ventricle is significantly reduced in ovariectomized rats, but returns to baseline with estrogen replacement [37]. We based our parameter value for $e_{AT1}$ and $e_{AT2}$ on these changes in receptor densities. AT1R-bound Ang II ([AT1R-AngII]) and AT2R-bound Ang II ([AT2R-AngII]) concentrations are modeled by


$$\begin{array}{c} \frac{d[AT1R-AngII]}{dt}=g_{AT1}^{-}(E)\cdot c_{AT1}[AngII]-\frac{\ln(2)}{h_{AT1}}[AT1R-AngII] \end{array}$$ (10)

$$\begin{array}{c} \frac{d[AT2R-AngII]}{dt}=g_{AT2}^{+}(E)\cdot c_{AT2}[AngII]-\frac{\ln(2)}{h_{AT2}}[AT2R-AngII] \end{array}$$ (11)

with $h_{AT1}$ and $h_{AT2}$ denoting the half-lives of AT1R-bound Ang II and AT2R-bound Ang II from [18]. $c_{AT1}$ and $c_{AT2}$ denote the binding rates of Ang II to AT1R and AT2R, respectively, as in [18]. $g_{AT1}^{-}$ and $g_{AT2}^{+}$ represent the impact of estrogen on the respective binding rate.

We chose parameter values for $e_{i}$ based on reported changes in the respective RAS levels in the literature as described above. The values for $e_{i}$ for each RAS component impacted by estrogen levels are listed in Table 1. Model simulations of the estrogen-RAS model in isolation are shown in S2 Appendix.

**Table 1:** Values of the parameters $e_{i}$ that determine the impact of estrogen on renin-angiotensin system components. The $e_{i}$ parameters are unitless.

Name	Value	Equation(s)	References
$e_{AGT}$	0.15	$g_{AGT}^{+}$, Eq. 6	[25]
$e_{renin}$	0.15	$g_{renin}^{-}$, Eq. 4	[24]
$e_{ACE}$	5.0	$g_{ACE}^{-}$, Eqs. 8, 9	[27]
$e_{AT1}$	0.5	$g_{AT1}^{-}$, Eqs. 9, 10	[38]
$e_{AT2}$	0.05	$g_{AT2}^{+}$, Eqs. 9, 11	[37]

#### RAS inhibitor simulations.

To simulate the RAS inhibitors, we use the same approach as in [18]. Specifically, to simulate treatment with an angiotensin receptor blocker (ARB), we let


$$\begin{array}{c} c_{AT1}=c_{AT1,0}\cdot (1-\sigma_{ARB}) \end{array}$$ (12)

where $c_{AT1,0}$ is the baseline value for $c_{AT1}$ and $\sigma_{ARB}$ is the percent inhibition of AT1R binding by the ARB. Similarly, to simulate treatment with an ACE inhibitor (ACEi), we let


$$\begin{array}{c} c_{ACE}=c_{ACE,0}\cdot (1-\sigma_{ACEi}) \end{array}$$ (13)

where $c_{ACE,0}$ is the baseline value for $c_{ACE}$ and $\sigma_{ACEi}$ is the percent inhibition of ACE by the ACEi.

### Calcium homeostasis and bone biology model

The mathematical model developed by Peterson and Riggs [13] was chosen as the basis for the calcium homeostasis and bone remodeling components of the composite model because it includes detailed bone, renal, and blood compartments, and predicts levels of PTH, calcitriol, bone cell activity, calcium, and phosphate. We further incorporated specific additions from published model extensions [14,16]:

- Gaweda et al. [16] extended the original model to include additional regulatory mechanisms governing calcium and phosphate homeostasis. In this study, we included the impact of phosphate levels on parathyroid gland capacity as added by Gaweda et al. [16] (equation for *H*_5,11_ in [16]). Note that Gaweda et al. [16] used the Peterson and Riggs [13] model as a basis for their extensions so all other parameters and biological components are the same as in [13].
- To evaluate long-term structural changes within the bone compartment, we incorporated the bone mineral density (BMD) compartment introduced by Riggs et al.[14].
- To investigate estrogen-driven changes in calcium homeostasis and bone remodeling, we integrated the estrogen effects added in Riggs et al.[14]. The authors included the impact of estrogen on latent and active TGF-β, osteoblast signaling, and renal calcium handling. TGF-β is a key cytokine in bone remodeling that regulates osteoblast and osteoclast proliferation and activity. Here, TGF-β refers to canonical transforming growth factor-β signaling as represented in the underlying Peterson–Riggs framework [13], rather than the broader TGF-β superfamily including bone morphogenetic proteins. Estrogen inhibits latent TGF-β formation, while stimulating its activation. TGF-β impacts bone remodeling activity through stimulating the expression of RANK on osteoclasts, stimulating osteoclast apoptosis, stimulating pre-osteoblast formation, and inhibiting osteoblast proliferation. Estrogen directly inhibits both the formation of pre-osteoblasts as well as the apoptosis rate of osteoblasts. Riggs et al. [14] also included the impact of estrogen on renal calcium reabsorption by decreasing renal calcium reabsorption with declining estrogen levels post-menopause. All of these estrogen effects are included in the model used in this study.

A model schematic is shown in Fig 1. The model equations are listed in S1 Appendix.

### Composite calcium homeostasis, bone remodeling, and RAS model

To formulate a composite model, we connected the estrogen-RAS model (Section Renin-angiotensin system model) with the calcium homeostasis and bone biology model (Section Calcium homeostasis and bone biology model) through known regulatory mechanisms. Specifically, the composite model includes the impact of AT1R-bound Ang II and calcium on PTH secretion (Section AT1R-bound Ang II and calcium impact on PTH secretion), the impacts of extracellular calcium levels and the calciotropic hormones on renin secretion (Section Calciotropic hormones, calcium, and renin secretion), and the impact of AT1R-bound Ang II on bone remodeling (Section AT1R-bound Ang II activation of RANKL and OPG).

To model those interactions, we follow the approach in [13] and characterize the changes in responses influenced by a given stimulant using hyperbolic functions:


$$\begin{array}{c} H_{x}^{-}=\alpha -(\alpha -\rho )\cdot \frac{x^{\gamma }}{\delta^{\gamma }+x^{\gamma }} \end{array}$$ (14)

$$\begin{array}{c} H_{x}^{+}=\rho +(\alpha -\rho )\cdot \frac{x^{\gamma }}{\delta^{\gamma }+x^{\gamma }} \end{array}$$ (15)

where *x* denotes the stimulus variable, α is the maximum anticipated response, γ is the steepness of the response (note when γ=1 the functions are sigmoidal), δ is the value of *x* that produces the half-maximal response, and ρ is the minimum anticipated response. The minus or plus superscript in *H* denotes whether the response is a decrease or increase from the baseline steady state, respectively. Note that when ρ=0, the response is a classical $E_{max}$ response often used in modeling in pharmacology (see [39]). These hyperbolic response functions were chosen because they provide a simple phenomenological representation of saturating physiological regulation with bounded responses. The parameters α, ρ, δ, and γ allow flexible control of maximal/minimal response, sensitivity, and response steepness while maintaining smooth nonlinear behavior. This formulation is also consistent with the approach used in the underlying Peterson and Riggs framework [13]. Parameter values for the equations added in this study are listed in Table 2. The newly introduced parameters in Tables 1, 2 represent the principal coupling (‘crosslinking’) parameters connecting the estrogen-RAS subsystem with the calcium homeostasis and bone remodeling subsystems. These parameters govern the strength and sensitivity of the inter-system regulatory interactions introduced in this study. Additional parameter values can be found in [13,14,16] or using our publicly available model code.

**Table 2:** Parameter values for coupling the RAS components to the calcium homeostasis and bone remodeling model. Additional parameters can be found in [13,14,16].

Name	Units	Value	Equation(s)	Reference(s)
$\alpha_{AT1R,PTH}$	–	3.2	Eq. 16	[40]
$\rho_{AT1R,PTH}$	–	0.7	Eq. 16	[40]
$\delta_{AT1R,PTH}$	pmol/L	10.0	Eq. 16	[40]
$\gamma_{AT1R,PTH}$	–	1.5	Eq. 16	[40]
$\alpha_{CaSR,PTH}$	–	320	Eq. 17	[40]
$\rho_{CaSR,PTH}$	–	155	Eq. 17	[40]
$\delta_{CaSR,PTH}$	pmol/L	2.4	Eq. 17	[40]
$\gamma_{CaSR,PTH}$	–	157	Eq. 17	[40]
$\alpha_{CaSR,renin}$	–	1.2	Eq. 19	[41,42]
$\rho_{CaSR,renin}$	–	0.25	Eq. 19	[41,42]
$\delta_{CaSR,renin}$	mmol/L	2.6	Eq. 19	[41,42]
$\gamma_{CaSR,renin}$	–	5.0	Eq. 19	[41,42]
$\alpha_{PTH,renin}$	–	2.55	Eq. 20	[40]
$\rho_{PTH,renin}$	–	0.55	Eq. 20	[40]
$\delta_{PTH,renin}$	pmol/L	4.0	Eq. 20	[40]
$\gamma_{PTH,renin}$	–	3.0	Eq. 20	[40]
$\alpha_{Ctriol,renin}$	–	1.1	Eq. 21	[43]
$\rho_{Ctriol,renin}$	–	0.8	Eq. 21	[43]
$\delta_{Ctriol,renin}$	pmol/L	180	Eq. 21	[43]
$\gamma_{Ctriol,renin}$	–	1.0	Eq. 21	[43]
$\alpha_{AT1R,RANKL}$	–	5.0	Eq. 23	[7]
$\rho_{AT1R,RANKL}$	–	0.95	Eq. 23	[7]
$\delta_{AT1R,RANKL}$	pmol/L	16.0	Eq. 23	[7]
$\gamma_{AT1R,RANKL}$	–	3.0	Eq. 23	[7]
$\alpha_{AT1R,OPG}$	–	2.5	Eq. 24	[7]
$\rho_{AT1R,OPG}$	–	0.9	Eq. 24	[7]
$\delta_{AT1R,OPG}$	pmol/L	14	Eq. 24	[7]
$\gamma_{AT1R,OPG}$	–	2.0	Eq. 24	[7]

#### AT1R-bound Ang II and calcium impact on PTH secretion.

AT1R-bound Ang II concentration, denoted by [AT1R-AngII], increases PTH secretion from the parathyroid gland [6]. To model this effect, we let $H_{AT1R,PTH}^{+}$ denote the impact of AT1R-bound Ang II on PTH secretion:


$$\begin{array}{c} H_{AT1R,PTH}^{+}=\rho_{AT1R,PTH}+(\alpha_{AT1R,PTH}-\rho_{AT1R,PTH})\cdot \frac{[AT1R-AngII{]}^{\gamma_{AT1R,PTH}}}{\delta_{AT1R,PTH}^{\gamma_{AT1R,PTH}}+[AT1R-AngII{]}^{\gamma_{AT1R,PTH}}}. \end{array}$$ (16)

where the parameters correspond to the function form of type Eq. 15.

Conversely, PTH secretion is inhibited by extracellular calcium levels ([Ca]) [44]. To model this effect, we let $H_{CaSR,PTH}^{-}$ be of the form of Eq. 14:


$$\begin{array}{c} H_{CaSR,PTH}^{-}=\alpha_{CaSR,PTH}-(\alpha_{CaSR,PTH}-\rho_{CaSR,PTH})\cdot \frac{[Ca{]}^{\gamma_{CaSR,PTH}}}{\delta_{CaSR,PTH}^{\gamma_{CaSR,PTH}}+[Ca{]}^{\gamma_{CaSR,PTH}}}. \end{array}$$ (17)

PTH concentration ([PTH]) in the blood is modeled by


$$\begin{array}{c} \frac{d[PTH]}{dt}=S_{PTH}\cdot H_{AT1R,PTH}^{+}\cdot H_{CaSR,PTH}^{-}-k_{deg-PTH}\cdot [PTH] \end{array}$$ (18)

where $S_{PTH}$ is the secretion of PTH from the parathyroid gland and $k_{deg-PTH}$ is degradation as in [13]. The parameters for Eqs. 16 and 17 were fit using the Ang II only experiment data from Grant et al. [40] (Section Parameter fitting to PTH and Ang II infusion experiments). The parameter values are listed in Table 2.

#### Calciotropic hormones, calcium, and renin secretion.

Renin secretion, denoted by $R_{sec}$, is impacted by [PTH], calcitriol ([Ctriol]), and [Ca]. Specifically, increased [Ca] and [Ctriol] inhibit renin secretion, while [PTH] stimulates renin secretion [6].

Extracellular calcium levels are detected by the calcium sensing receptor (CaSR) in the juxtaglomerular cells [45]. To model the impact of CaSR on $R_{sec}$, we let:


$$\begin{array}{c} H_{CaSR,renin}^{-}=\alpha_{CaSR,renin}-(\alpha_{CaSR,renin}-\rho_{CaSR,renin})\cdot \frac{[Ca{]}^{\gamma_{CaSR,renin}}}{\delta_{CaSR,renin}^{\gamma_{CaSR,renin}}+[Ca{]}^{\gamma_{CaSR,renin}}}. \end{array}$$ (19)

Ortiz-Capisano et al. [41,42] found that renin release was 40% lower when using a calcimimetic or at elevated calcium levels in the juxtaglomerular cells of rat kidneys. We used this result to estimate parameters so that at high calcium levels, renin release is decreased by 40%.

To model stimulation of $R_{sec}$ by [PTH], we let:


$$\begin{array}{c} H_{PTH,renin}^{+}=\rho_{PTH,renin}+(\alpha_{PTH,renin}-\rho_{PTH,renin})\cdot \frac{[PTH{]}^{\gamma_{PTH,renin}}}{\delta_{PTH,renin}^{\gamma_{PTH,renin}}+[PTH{]}^{\gamma_{PTH,renin}}}. \end{array}$$ (20)

The parameters (listed in Table 2) were fit using the PTH only infusion experiment from Grant et al. [40] (Section Parameter fitting to PTH and Ang II infusion experiments).

To model inhibition of $R_{sec}$ by calcitriol, we let:


$$\begin{array}{c} H_{Ctriol,renin}^{-}=\alpha_{Ctriol,renin}-(\alpha_{Ctriol,renin}-\rho_{Ctriol,renin})\cdot \frac{[Ctriol{]}^{\gamma_{Ctriol,renin}}}{\delta_{Ctriol,renin}^{\gamma_{Ctriol,renin}}+[Ctriol{]}^{\gamma_{Ctriol,renin}}}. \end{array}$$ (21)

The parameters were selected to produce a physiologically consistent inhibitory relationship between calcitriol and renin secretion guided by the trends reported in Tomaschitz et al. in human participants [43]. Taken together, we incorporate the combined impact of calcium, PTH, and calcitriol on renin secretion by modifying Eq. 4 to


$$\begin{array}{c} R_{sec}=N_{rs}\cdot \nu_{AT1R}\cdot g_{renin}^{-}(E)\cdot H_{CaSR,renin}^{-}\cdot H_{PTH,renin}^{+}\cdot H_{Ctriol,renin}^{-}. \end{array}$$ (22)

#### AT1R-bound Ang II activation of RANKL and OPG.

Shimizu et al. [7] reported that in ovariectomized rats, infusion of Ang II increased RANKL expression and led to a significant decrease in bone volume, but this impact was abolished by treatment with an AT1R blocker and not an AT2R blocker. Their results showed that RANKL production is activated by AT1R-bound Ang II levels. Additionally, Shimizu et al. [7] found that OPG expression is also increased in osteoblast cells due to infusion of Ang II, but to a much lesser extent than RANKL.

The impact of AT1R-bound Ang II on RANKL and OPG expression is modeled using the form in Eq. 15 for activation so that:


$$\begin{array}{c} H_{AT1R,RANKL}^{+}=\rho_{AT1R,RANKL}+(\alpha_{AT1R,RANKL}-\rho_{AT1R,RANKL})\cdot \frac{[AT1R-AngII{]}^{\gamma_{AT1R,RANKL}}}{\delta_{AT1R,RANKL}^{\gamma_{AT1R,RANKL}}+[AT1R-AngII{]}^{\gamma_{AT1R,RANKL}}}, \end{array}$$ (23)

$$\begin{array}{c} H_{AT1R,OPG}^{+}=\rho_{AT1R,OPG}+(\alpha_{AT1R,OPG}-\rho_{AT1R,OPG})\cdot \frac{[AT1R-AngII{]}^{\gamma_{AT1R,OPG}}}{\delta_{AT1R,OPG}^{\gamma_{AT1R,OPG}}+[AT1R-AngII{]}^{\gamma_{AT1R,OPG}}}. \end{array}$$ (24)

These are included in the equations for the production of RANKL and OPG, respectively (Eqs. F.6 and F.8 in S1 Appendix). The values for $\alpha_{AT1R,RANKL}$ and $\alpha_{AT1R,OPG}$ represent the highest possible amount of activation of RANKL and OPG by AT1R binding, respectively. These values were set to the fold-change in RANKL and OPG expression after Ang II infusion in Shimizu et al. [7]. The parameter values are listed in Table 2.

#### Parameter fitting to PTH and Ang II infusion experiments.

Grant et al. [40] conducted infusion experiments in healthy volunteers to determine the relationship between the RAS and PTH in humans. To test this, the authors gave healthy volunteers 1 hour infusions on different days of (i) Ang II only, (ii) PTH (i.e., teriparatide) only, and (iii) Ang II and PTH together. We simulated these experiments and used the data from this study to determine parameters values (listed in Table 2) for Eqs. 16, 17, and 20.

In their experiments, Ang II infusions were given in graded doses of 1, 3, and 10 ng/kg·min for consecutive infusions of 20 minutes each. We simulated Ang II infusion by letting $k_{inf}=1$ ng/kg·min (Eq. 9) for the first 20 minutes, 3 ng/kg·min for the next 20 minutes, and 10 ng/kg·min for the final 20 minutes of the 1 hour infusion to simulate this infusion type. For PTH infusions we used the pharmacokinetic parameters from Peterson and Riggs [13] with a dose of 200 U of teriparatide as reported by Grant et al. [40]. To fit the parameters, we used the fmincon function from MATLAB using the interior-point algorithm to minimize the difference between the predicted values and the data for PRA, intact PTH, and [Ang II] reported in Grant et al. [40]. That is, parameter values were determined by minimizing the summed squared error between model predictions and the experimental data points. In all of these simulations we let *E* = 1 so that there are no estrogen effects.

### Sobol sensitivity analysis

Mathematical models of complex biological systems, like the one used in this study, involve a large number of parameters with unknown or uncertain values. To investigate the impact of variations in parameter values on model output, we can use methods such as sensitivity analysis [46,47]. The Sobol analysis was also used to assess the robustness of model outputs to uncertainty and variability in parameter values. Global sensitivity analysis methods evaluate the model at many points in the parameter space, providing an assessment of how individual parameters contribute to variations in model output across the parameter space, including interactions with other parameter changes [46–48].

The Sobol method is a variance-based global sensitivity analysis technique that decomposes the variance in the model output into contributions from each individual parameter as well as its interactions with other parameters [46,49]. The results of a Sobol sensitivity analysis are called “Sobol sensitivity indices” (or “Sobol indices” for short) and are computed for each parameter included in the analysis. First-order Sobol indices (denoted by $S_{first}$) are the fraction of the variance in the model output the individual parameter contributes to. Total order Sobol indices (denoted by $S_{total}$) quantify the fraction of output variance the parameter contributes to, including interactions with other parameter changes. By evaluating Sobol indices, we can identify which parameters are the most influential on model output. For more information about Sobol sensitivity analysis and its use in applications in biology, we refer readers to [46–49].

We conducted a Sobol sensitivity analysis using simulations of the full composite model (described in Section Composite calcium homeostasis, bone remodeling, and RAS model). The model outputs we considered were bone mineral density (Eq. F.13 in S1 Appendix), AT1R-bound Ang II (Eq. 10), PTH (Eq. 18), calcitriol (Eq. D.3 in S1 Appendix), extracellular calcium (Eq. B.1 in S1 Appendix), and renin concentration (Eq. 5) over a simulation time from 20 to 80 years with age-related estrogen decline. We computed Sobol indices at every 10 years of simulation time.

The full composite model contains 150 parameters, making a Sobol sensitivity analysis over the full parameter set computationally expensive and prone to numerical instability. To make the analysis computationally tractable, we used a two-step procedure.

First, we conducted a Sobol sensitivity analysis varying the 121 parameters belonging to the calcium homeostasis and bone remodeling subsystem by ±10%. These parameters included those governing bone remodeling, PTH secretion and degradation, calcitriol synthesis and degradation, renal calcium and phosphate handling, intestinal calcium and phosphate absorption, and extracellular calcium and phosphate levels. Parameters with first-order Sobol indices $S_{first}>0.01$ for at least one output variable at any simulation time point were retained. This screening step identified 30 sensitive calcium/bone subsystem parameters.

Second, we combined these 30 retained parameters with all newly introduced parameters from Tables 1 and 2, including all estrogen-RAS and RAS-calcium-bone coupling parameters, yielding 59 parameters in the final Sobol sensitivity analysis. Thus, no newly introduced coupling parameters were excluded during the screening step. The final Sobol analysis varied parameters by ±25% of baseline values. The results of the Sobol sensitivity analysis are described in the Sensitivity analysis section.

## Results

### PTH and Ang II infusion experiments simulations

We conducted simulations of infusion of (i) Ang II, (ii) PTH, and (iii) PTH and Ang II together (described in the Parameter fitting to PTH and Ang II infusion experiments section). Model results, together with the experimental data reported for the clinical studies done by Grant et al. [40], are shown in Fig 2.

**Fig 2:**
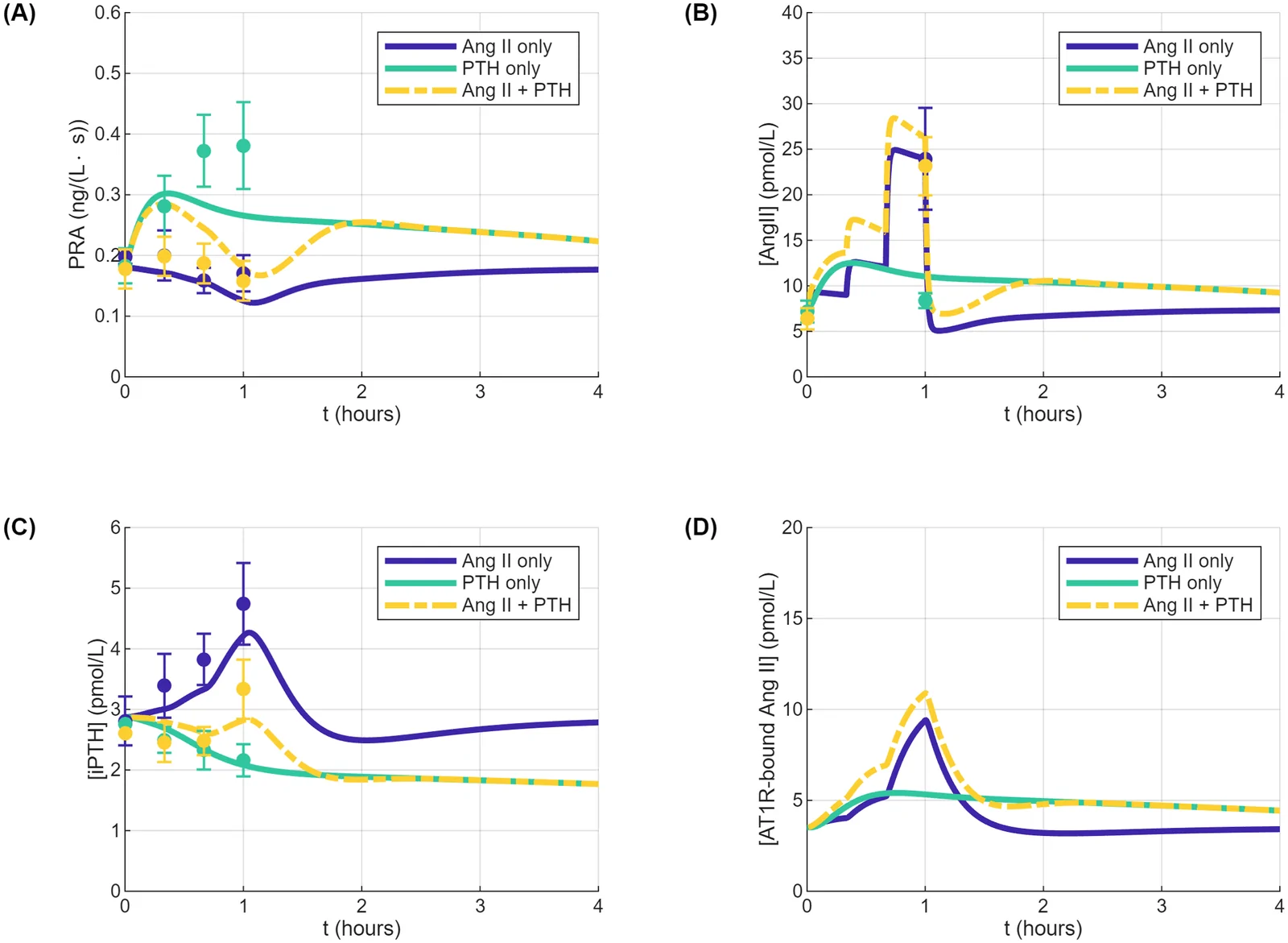
Simulation results for infusion of Ang II (“Ang II only”), PTH (“PTH only”), and Ang II and PTH together (“Ang II + PTH”). Shown are trajectories for (A) plasma renin activity, (B) angiotensin II plasma concentration, **(C)**, intact parathyroid hormone plasma concentration, and (D) angiotensin receptor type 1-bound Ang II during and following the 1 hour infusion period. Lines indicate simulations, while the dots and error bars in panels (A–C) are from the relevant experimental data presented in Grant et al. [40]. Ang II: angiotensin II, AT1R: angiotensin receptor type 1, iPTH: intact parathyroid hormone, PRA: plasma renin activity.

When Ang II is infused in isolation, Ang II levels rise, resulting in an increase in AT1R-bound Ang II levels as receptor occupancy increases in response to the higher circulating ligand concentration (Fig 2(B), 2(D)). This stimulates PTH secretion so that endogenous PTH (intact PTH, denoted by [iPTH]) increases (Fig 2(C)). Grant et al. [40] reported that during an Ang II infusion, [iPTH] increased, while PRA decreased slightly likely due to the negative feedback on renin secretion by AT1R-bound Ang II (Fig 2 “Ang II only” data). Model simulations capture both the rise in [iPTH] (Fig 2(C) “Ang II only”) and slight decrease in PRA (Fig 2(A) “Ang II only”) during infusion.

When infusing PTH in healthy volunteers, PRA nearly doubled on average (Fig 2(A) “PTH only” data) [40]. Model simulations predicted a 69% increase in PRA (Fig 2(A) “PTH only”). This difference may reflects the absence of FGF-23 in the model. PTH increases circulating FGF-23 levels [50], which subsequently suppresses calcitriol, thereby increasing renin secretion [51]. Including FGF-23 effects could help strengthen and sustain the PTH induced renin increase. While total PTH levels increased substantially during the PTH infusion experiment, endogenous PTH production steadily decreased. This effect is captured by the model (Fig 2(C) “PTH only” data).

When infusing both Ang II and PTH together in healthy volunteers, the authors reported that Ang II levels were similar to those reported in the Ang II only experiment, likely due to PTH having only a small impact on Ang II levels [40]. In our model simulations, Ang II levels were slightly higher in the Ang II and PTH infusion experiment than the Ang II only experiment, but were still within the range reported by Grant et al. [40] at the end of the 1 hour infusion time (Fig 2(B) “Ang II + PTH”). Grant et al. [40] reported that PRA levels remained near baseline for the Ang II and PTH infusion experiment. In our model simulations, the “Ang II + PTH” simulations resulted in a transient increase in PRA but declines to near the levels prior to infusion by the end of the simulation time (Fig 2(A)). This discrepancy may stem from unmodeled PTH receptor dynamics. In vivo, Ang II suppresses renin release by binding to juxtaglomerular AT1R, while PTH stimulates renin release by binding to juxtaglomerular parathyroid hormone receptor 1 (PTH1R) [52]. However, the current model lacks PTH receptors and instead assumes that PTH directly stimulates renin secretion, whereas Ang II must first undergo receptor binding. Consequently, the stimulation by PTH is faster acting, which may resulting in the observed transient increase in renin secretion before it is counteracted by the AT1R-bound Ang II mediated inhibition.

### Impact of estrogen decline and RAS inhibitors

How do declining estrogen levels in post-menopausal women impact the RAS? How do changes in the RAS and declining estrogen levels contribute to altered bone remodeling? Does bone mineral density improve with RAS inhibition? To investigate these questions, we conducted simulations of the composite calcium homeostasis, bone remodeling, and estrogen-RAS model with estrogen decline observed in post-menopausal women with and without drug interventions.

In each simulation, estrogen decline starts at 50 years, which is about the average age of menopause onset in women. We conducted simulations with key RAS inhibitors (ACEi and ARB) starting at age 60 years, unless otherwise indicated. For ARB simulations, we let $\sigma_{ARB}$ (Eq. 12) vary from 88–98.3% as reported for different types and typical dose levels of ARBs by Hallow et al. [18]. For ACEi, we conducted simulations with $\sigma_{ACEi}$ (Eq. 13) varying from 94–97.2% as reported for common types of ACEi and doses by Hallow et al. [18]. The results are shown in Figs 3 and 4.

**Fig 3:**
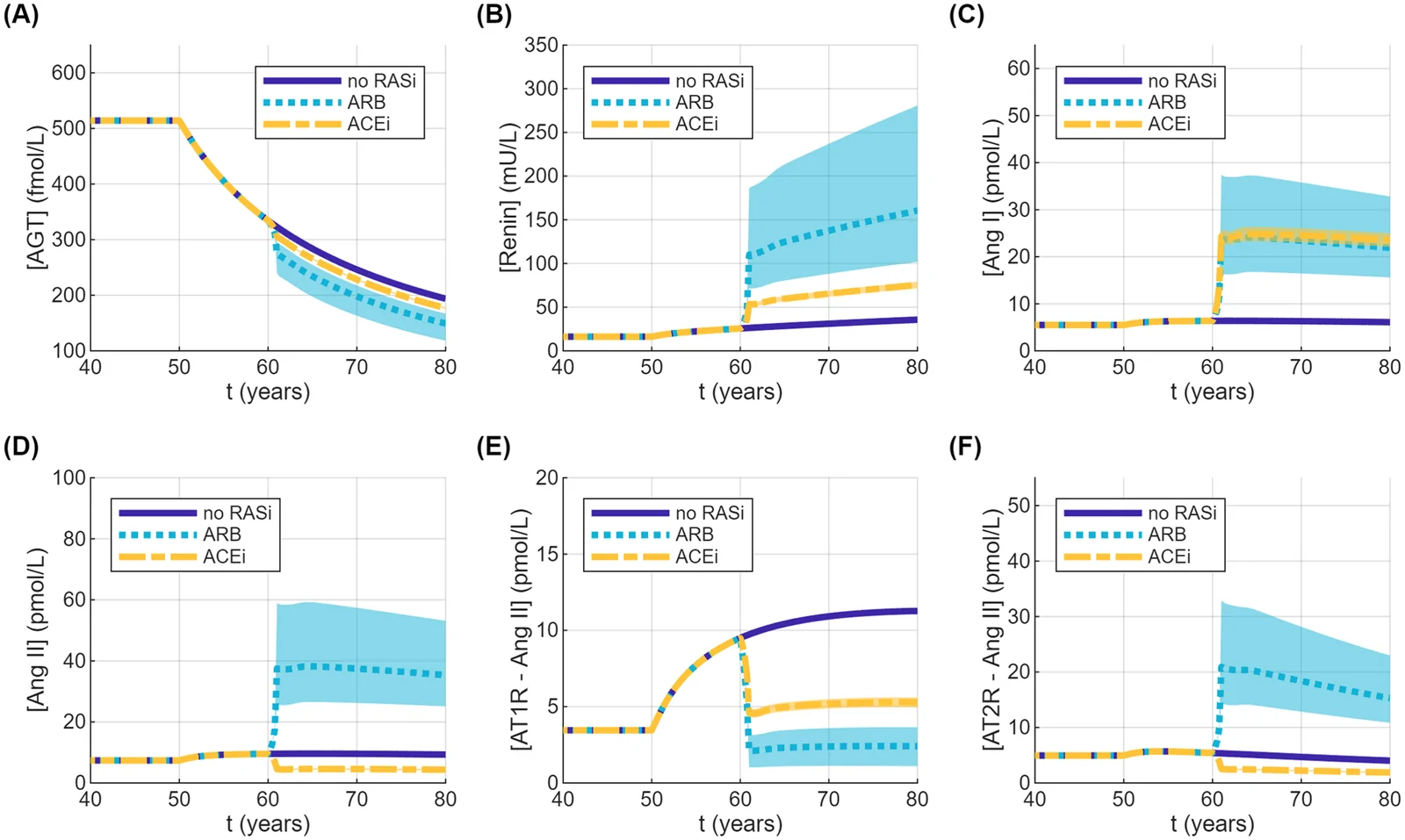
Impact of post-menopausal estrogen decline and renin-angiotensin system inhibitors on the renin-angiotensin system. For the angiotensin receptor blocker simulations (labeled “ARB” in the legend), $\sigma_{ARB}$ (Eq. 12) was varied between 88–98.3% based on the ranges reported in [18]. For the angiotensin-converting enzyme inhibitor simulations (labeled “ACEi” in the legend), $\sigma_{ACEi}$ (Eq. 13) was varied between 94–97.2% as reported in [18]. Lines represent the mean values over the varied $\sigma_{i}$ values for the respective treatment with shaded area representing the range. AGT: angiotensinogen, Ang I: angiotensin I, Ang II: angiotensin II, AT1R: angiotensin type 1 receptor, AT2R: angiotensin type 2 receptor, RAS: renin angiotensin system, RASi: RAS inhibitor, ARB: angiotensin receptor blocker, ACEi: angiotensin-converting enzyme inhibitor.

**Fig 4:**
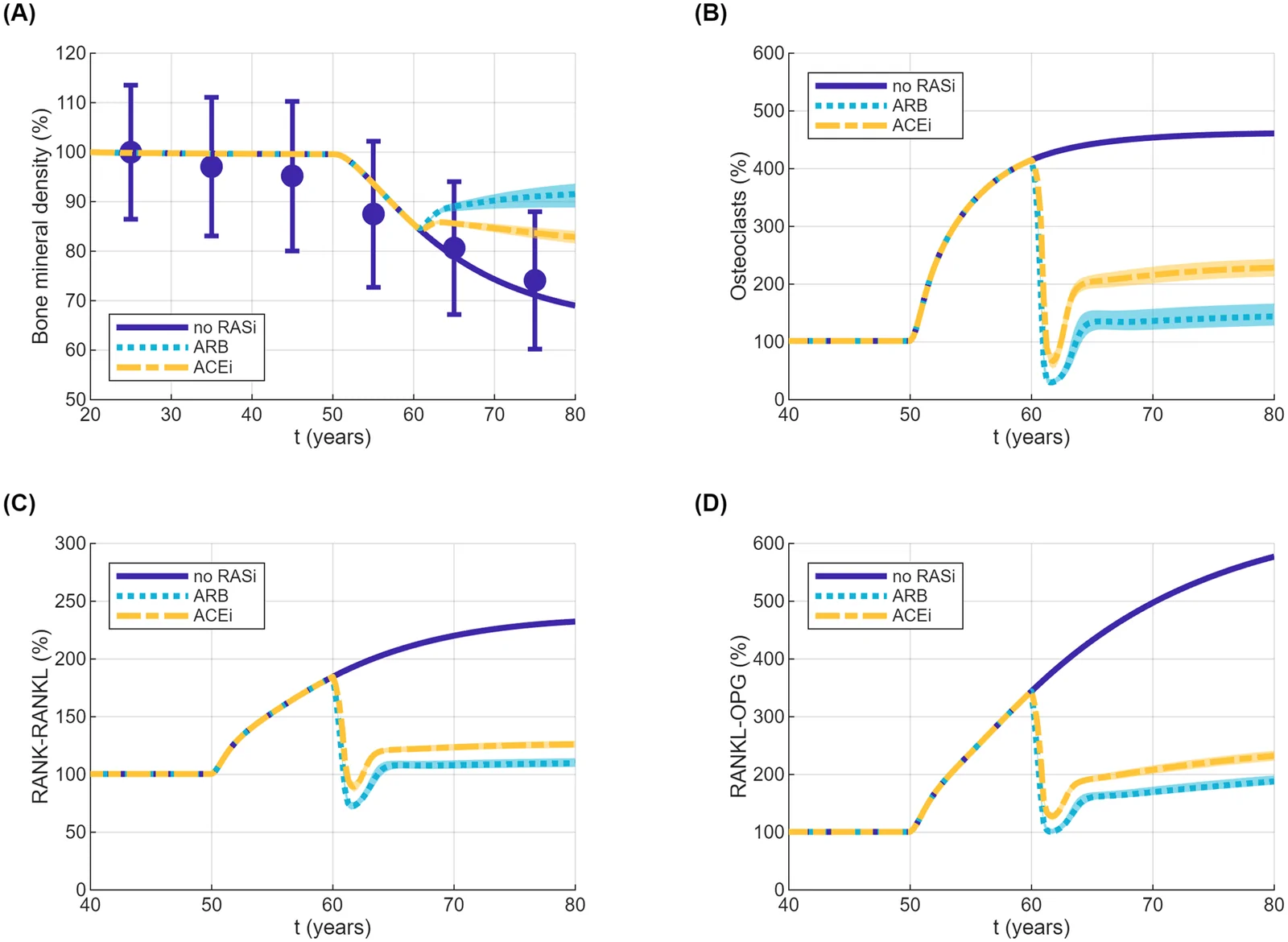
Impact of post-menopausal estrogen decline and RAS inhibitors on (A) bone mineral density, (B) osteoclast levels, (C) RANK-RANKL levels, and (D) RANK-OPG levels. The values are normalized to their baseline levels and shown as a percent of the baseline value. For the angiotensin receptor blocker simulations (labeled “ARB” in the legend), $\sigma_{ARB}$ (Eq. 12) was varied between 88–98.3% based on the ranges reported in [18]. For the angiotensin-converting enzyme inhibitor simulations (labeled “ACEi” in the legend), $\sigma_{ACEi}$ (Eq. 13) was varied between 94–97.2% as reported in [18]. Dashed lines represent the mean values over the varied $\sigma_{i}$ values for the respective treatment, with shaded areas representing the corresponding ranges. Data in panel (A) are from the female data in Looker et al. [53]. Both simulations and data are for femoral neck bone mineral density. BMD: bone mineral density, RANK: receptor activator of NF-kappaB, RANKL: RANK ligand, OPG: osteoprotegerin, RASi: RAS inhibitor, ARB: angiotensin receptor blocker, ACEi: angiotensin-converting enzyme inhibitor.

ACEi and ARB inhibit ACE activity and AT1R binding, respectively. While these are different components of the RAS, both drugs result in reduced levels of AT1R-bound Ang II (Fig 3(E)). The impact of these drugs on the various components of the RAS are drug and dose-dependent. The varied range in values of $\sigma_{ACEi}$ had a negligible effect on our model simulation results (Fig 3, “ACEi”). In the ARB simulations, the impact on the RAS has a wider variation based on the value of $\sigma_{ARB}$ (Fig 3, “ARB”). Note that we simulated a wider range for $\sigma_{ARB}$ than $\sigma_{ACEi}$.

We first consider the impact of ACEi and ARB on the RAS components. ARB directly inhibits the binding of Ang II to AT1R. As a result, AT1R-bound Ang II levels decreased by about 66–89%, depending on efficacy (Fig 3(E), “ARB” vs. “no RASi”). Recall that AT1R-bound Ang II inhibits renin secretion (Fig 1). Hence, decreased AT1R-bound Ang II levels result in increased renin levels (Fig 3(B)) as well as increased downstream Ang I (Fig 3(C)) and Ang II levels (Fig 3(D)). The lower AT1R binding along with increased Ang II levels results in more Ang II binding to AT2R, so that AT2R-bound Ang II levels increase by about 4-fold on average (Fig 3(F) “ARB” vs. “no RASi”’). ACEi impact the RAS by inhibiting the activity of ACE, which is the enzyme that converts Ang I into Ang II (Fig 1). Ang II levels decreased by an average of 52% in the ACEi simulations despite increased Ang I levels (Fig 3(D), 3(C) “ACEi” vs. “no RASi”). As a result, both AT2R-bound Ang II and AT1R-bound Ang II decreased by about 52% from the no RAS inhibitor case (Fig 3(E), 3(F), “ACEi” vs. “no RASi”).

Next, we analyze the age-related estrogen decline effect (S2 Appendix) on bone mineral density and its response to ACEi and ARB. Estrogen maintains bone density by regulating factors involved in bone remodeling that promote the differentiation and activation of osteoblasts, while inhibiting osteoclast activity (Fig 1). When estrogen levels decline, osteoclast activity increases, which increases bone resorption, ultimately resulting in a decrease in bone mineral density (Fig 4(A), 4(B) “no RASi”). The bone mineral density simulations results with the decline in estrogen levels is consistent with the femoral neck BMD data reported by Looker et al. for age-stratified women, as measured by dual-energy X-ray absorptiometry [53] (Fig 4(A) “no RASi” data). This decrease is a result of a combination of the direct impacts of estrogen on bone remodeling and increased AT1R-bound Ang II levels, which stimulates RANKL production to bind with RANK (Fig 4(C); Fig 3(E) “no RASi”). RANK-RANKL stimulates osteoclast differentiation and activation. Throughout the estrogen decline simulations, the calciotropic hormones, PTH and calcitriol, as well as key electrolytes, calcium and phosphate, remained within normal range (Fig B in S3 Appendix).

Age-related decreases in estrogen levels result in a 4.5-fold increase in osteoclast activity (Fig 4(B) “no RASi”). However, with ARB, AT1R-bound Ang II levels decrease (Fig 3(E)), resulting in reduced RANKL production, so that osteoclast levels return to about 40% above baseline levels (Fig 4(B), 4(C)). As a result, by the end of the simulation time (i.e., *t* = 80 years), bone mineral density is at about 90% of baseline for ARB users versus 75% before estrogen decline with no treatment (Fig 4(A) “ARB” vs. “no RASi”). ACEi also decreased AT1R-bound Ang II levels by a lesser amount (Fig 3(E)) so that osteoclast levels are lowered to about 2-fold baseline levels. This results in a slight improvement in bone mineral density to about 85% of the level before estrogen decline (Fig 4(A) “ACEi”). We observed that the range in the amount of AT1R-bound Ang II inhibition due to varied ARB efficacy did not have a large impact the change in bone levels on these drugs (Fig 3, Fig 4). Thus, the improvement to bone mineral density may not depend significantly on the dose of the RAS inhibitors within their typically prescribed ranges. This does not indicate a lack of dose dependence in the BMD response, but rather that the dependence on dose is not significant within the pharmacologically relevant RAS inhibition ranges.

We conducted simulations of RAS inhibitor treatment starting at different ages with age-related estrogen decline as in Eq. 1. In these simulations, we let $\sigma_{ARB}=93\%$ and $\sigma_{ACEi}=95\%$ based on the average value for the respective drugs from [18]. We did this because varying $\sigma_{i}$ did not have much impact on bone mineral density (Fig 4(A)). The results are shown in Fig 5. In all simulations, bone mineral density improved on both the ARB and ACEi drugs with a greater improvement with ARB treatment. When the administration of ARB or ACEi was started before the onset of estrogen decline (i.e., starting a RAS inhibitor before 50 years old), bone mineral density increased from baseline by about 5% (Fig 5(A), 5(C). When estrogen decline started at 50 years, the bone mineral density decreased, but stays above 95% for patients on ARB (Fig 5(A)). When the administration of ACEi began before estrogen decline, bone mineral density declined below 90% of baseline during age-related estrogen decline (Fig 5(C)). Our model simulations suggest that early RAS inhibition prevents bone loss driven by age-related estrogen decline.

**Fig 5:**
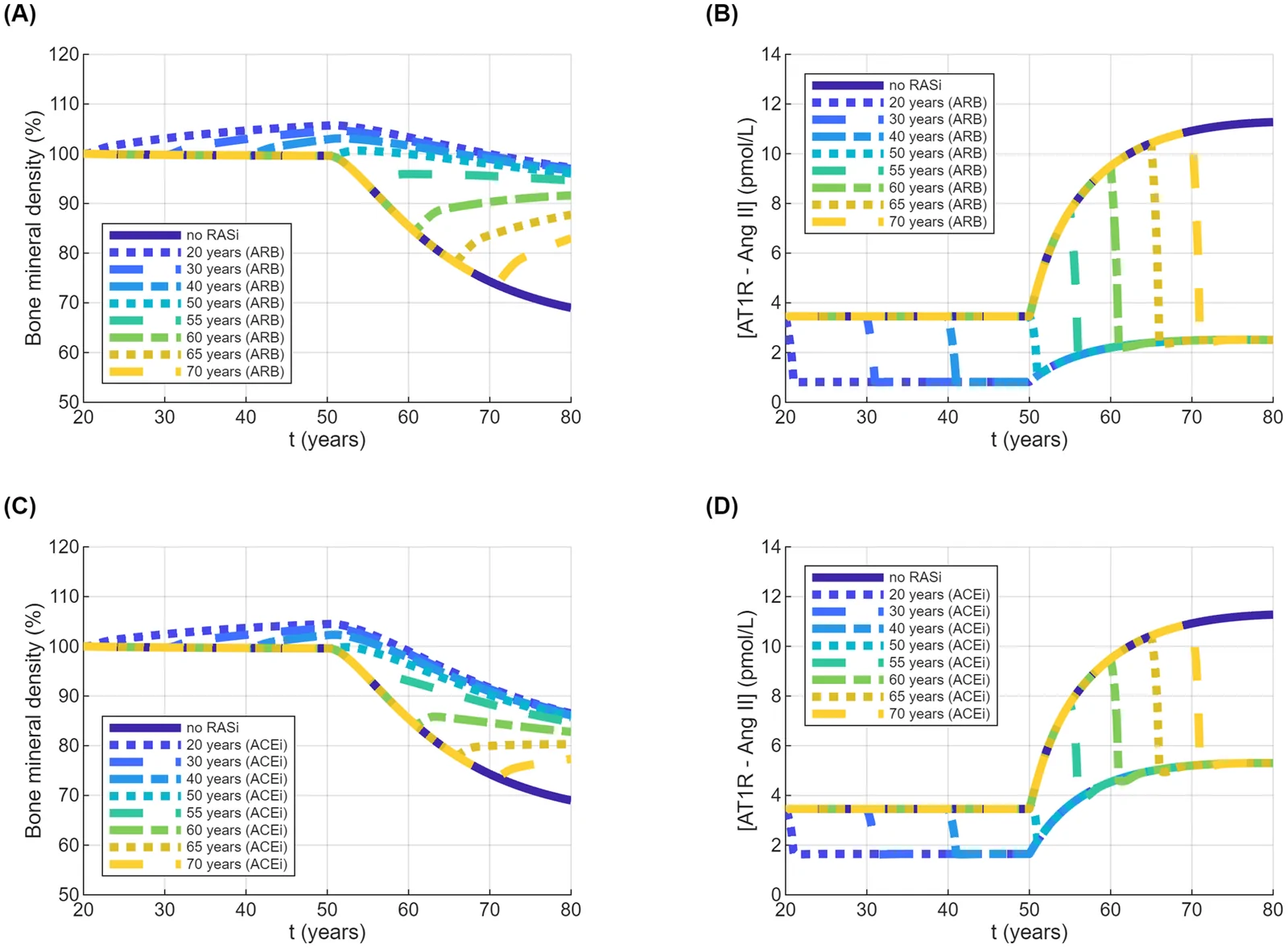
Model simulation results for varied starting age of angiotensin receptor blocker (A–B) and angiotensin-converting enzyme inhibitor (C–D). $\sigma_{ARB}=93\%$ in all simulations for ARB and $\sigma_{ACEi}=95.6\%$ in all ACEi simulations. The bone mineral density is femoral neck bone mineral density. AT1R: angiotensin type 1 receptor, ARB: angiotensin receptor blocker, ACEi: angiotensin-converting enzyme inhibitor, RASi: RAS inhibitor.

In summary, the RAS, calcium, and bone remodeling model developed in this study represents the expected decrease in bone mineral density due to age-related estrogen decline. This is driven by both the direct impacts of estrogen on bone remodeling and calcium regulation, as well as RAS activation by estrogen decline. Model simulations suggest that ARBs offer stronger protection for bone health than ACEi, and that result is consistent when starting treatment at different ages.

### Sensitivity analysis

We conducted a Sobol sensitivity analysis for the full composite model during age-related estrogen decline in women (see Sobol sensitivity analysis section for details). The parameters with the largest average $S_{total}$ values in Fig C(A) in S3 Appendix are the parameters that have the largest impact on variation in bone mineral density. The top five parameters, based on the average $S_{total}$ value, are all related to the regulation of RANK and RANKL (Fig C(A) in S3 Appendix). The RANK-RANKL complex directly impacts bone remodeling by stimulating osteoclasts (Fig 1). Specifically, the parameters $k_{in,L}$ and $k_{in,RNK}$ are baseline production rates of RANKL and RANK, respectively. The parameter $E_{max,L-PTH}$ relates to the impact of PTH on RANKL production. The parameters *k*_3_ and *k*_4_ are the binding and unbinding rate of the RANK-RANKL complex, respectively. The next parameter with the largest impact on bone mineral density is $\rho_{AT1R,L}$, which represents the minimum impact of AT1R on RANKL production (Eq. 23), thereby linking the RAS to bone remodeling. $\delta_{CaSR,renin}$ represents the sensitivity of renin secretion to calcium levels. This is the parameter with the largest impact on [AT1R-bound Ang II] (Fig C(B) in S3 Appendix).

The newly introduced parameters in Tables 1 and 2 represent the principal coupling (‘crosslinking’) parameters connecting the estrogen-RAS subsystem with the calcium homeostasis and bone remodeling subsystems. These parameters govern the strength and sensitivity of the inter-system regulatory interactions introduced in this study. In summary, the top parameters that impact bone mineral density all primarily relate to RANK, RANKL and RANK-RANKL binding.

AT1R-bound Ang II is the key biological component that connects the RAS with the calcium homeostasis and bone remodeling model (Fig 1). The parameters with the 5 largest average $S_{first}$ and $S_{total}$ values for the model output of [AT1R-bound Ang II] are $\delta_{CaSR,renin}$, $\Phi_{Plas2BoneCa0}$, $N_{rs}$, $c_{AT1R}$, and $X_{PRCPRA}$ (Fig C(B) in S3 Appendix). $\delta_{CaSR,renin}$ represents half-maximal response of renin secretion to calcium levels, linking the calcium regulatory system to the RAS. $\Phi_{Plas2BoneCa0}$ represents the baseline fluxes of calcium from the plasma to the bone, impacting variation in calcium and renin levels (Fig D in S3 Appendix). Calcium level changes affect the RAS via the CaSR in the renin-secreting juxtaglomerular cells of the kidneys. $N_{rs}$, $c_{AT1R}$, and $X_{PRCPRA}$ are all directly related to the RAS (Section Impact of estrogen decline in aging women on the RAS). Specifically, $N_{rs}$ determines baseline renin secretion, $c_{AT1R}$ is the binding rate for AT1R, and $X_{PRCPRA}$ is baseline conversion rate from renin concentration to activity. Our sensitivity analysis shows that variations in [AT1R-bound Ang II] are driven by the CaSR impact on renin secretion and the RAS cascade.

The calciotropic hormones are the primary regulators of calcium and bone remodeling. Additionally, these hormones have a bidirectional relationship with the RAS (Fig 1). In our Sobol sensitivity analysis, the parameters with the largest impact on PTH were $k_{base,AOH}$, $V_{max,PTH-AOH}$, $V_{max,Phos-AOH}$, and $\delta_{AOH,Phos}$ (Fig E(A) in S3 Appendix). $V_{max,PTH-AOH}$ represents the maximal stimulation by PTH on 1α-hydroxylase, the enzyme that synthesizes calcitriol. $V_{max,Phos-AOH}$ and $\delta_{AOH,Phos}$ characterize the maximal effect and half-maximal concentration for phosphate mediated calcitriol inhibition. PTH and calcitriol regulate one another, as calcitriol inhibits PTH secretion and PTH stimulates calcitriol production (Fig 1). Both PTH and calcitriol are sensitive to the parameter $k_{base,AOH}$ which is the baseline production rate of 1α-hydroxylase, and to the parameters $\Phi_{Bone2PlasCa0}$ and $\Phi_{Plas2BoneCa0}$, which denote the baseline rate of calcium exchange between the bone and plasma and vice versa (Fig E in S3 Appendix). The latter is likely due to the sensitivity of PTH to calcium levels, which are impacted by these two parameters as well (Fig D(A) in S3 Appendix).

While TGFβ is an important regulator of osteoblast and osteoclast activity in the model, parameters associated with TGFβ signaling did not rank among the most influential Sobol indices for the outputs considered here. This does not imply that TGFβ is physiologically unimportant; rather, within the parameter ranges and simulation conditions studied, variation in RANK/RANKL regulation and calcium homeostasis contributed more strongly to output variance.

The Sobol sensitivity analysis results shown here are the average (bar) and standard deviation (error bar) of $S_{first}$ and $S_{total}$ values over the full simulation time from age 20–80 years. We also analyzed the results over the simulation time from age 20–50, i.e., before estrogen decline. The parameters with the largest impact on model output were the same as those for the full simulation time. This suggests that the dominant sources of output variance arise from parameters governing the core calcium, RAS, and bone remodeling pathways, whereas the parameters mediating estrogen-dependent regulation have comparatively smaller contributions to global output variance within the parameter ranges considered.

This does not indicate that estrogen has little physiological influence in the model. Rather, it indicates that the model outputs are relatively less sensitive to the parameters mediating the estrogen impacts than to parameters governing the core RAS, bone and calcium pathways. During the menopause simulations used in the Sobol analysis, estrogen declines significantly over time, producing a substantial system-wide effect. The presence of estrogen decline itself is the dominant driver of the postmenopausal changes, while variation in the parameters controlling the precise shape of the estrogen impact produces comparatively smaller changes in model output at the global scale considered in the Sobol analysis.

In summary, our global sensitivity analysis shows that, in our model, bone remodeling is most sensitive to RANK-RANKL binding and that the RAS is sensitive to both calcium levels and the calciotropic hormones. These are the key biological components that impact RAS levels and bone remodeling.

## Discussion

In this study we developed a mathematical model that includes the interconnected systems of estrogen, the RAS, PTH, calcitriol, calcium regulation, and bone remodeling. We used this model to:

- Investigate the impact of estrogen decline, as experienced in post-menopausal women, on the RAS, bone remodeling, and calcium homeostasis.
- Simulate potential therapeutic effects of ACEi and ARB on bone remodeling and calcium regulation.
- Assess the sensitivity of key model outputs to variations in the model’s many unknown parameters using a Sobol sensitivity analysis.

To the best of our knowledge, this is the first model to incorporate the direct effects of estrogen on the RAS, as well as the integrated effects of the RAS on calcium regulatory and bone remodeling systems. As such, our model development and analysis have brought major advances to the active area of using mathematical modeling to investigate bone remodeling and calcium regulation.

Given that the prevalence of hypertension increases markedly with aging, hypertension and osteoporosis often coexist. RAS inhibitors are widely used, safe, reasonably priced long term treatments for hypertension. RAS inhibitors have also been proposed as a treatment to improve bone density due to the relationship between the RAS and bone remodeling [10,11]. Since RAS inhibitors are already widely used for the treatment of hypertension in aging populations, including post-menopausal women, it may be beneficial to consider which RAS inhibitor may prevent or improve bone loss.

We conducted model simulations to investigate the impacts of RAS inhibitors, specifically ACEi and ARB, on bone remodeling for various stages of age-related estrogen decline. Since treatment with an ARB results in a larger reduction in AT1R-bound Ang II for all dose levels, the improvement in bone mineral density was greater in simulations of ARB than ACEi (Fig 3(E), Fig 4(A)). It seems that the targeted impact of ARB on AT1R binding results in a better improvement in bone mineral density during post-menopausal estrogen decline than ACEi, which inhibit Ang II production via ACE. These results were consistent when we varied the starting age for ACEi and ARB before and after estrogen decline (Fig 5). In summary, our model simulations showed that reductions in AT1R-bound Ang II abundance results in reduced osteoclast activity, which prevents and improves declines in bone mineral density driven by low estrogen levels.

In a clinical study, Kim et al. [10] reported that patients that have used ARB only experienced fewer fractures than patients who have never used a RAS inhibitor. Similarly, Solomon et al. [54] reported reduced fracture risk in users of ARB, while ACEi users had no change in fracture risk. The systemic review by Wu et al. [12] reported that lower fracture risk is consistently reported in users of ARB, but not ACEi in men. In contrast, our mathematical model predicted that bone mineral density is increased not only by ARB treatment but ACEi as well (Fig 4(A)). That discrepancy may be due to mechanisms not captured by our model. It is possible that the improvement in bone mineral density predicted by our model for ACEi is not enough to have a notable impact on fracture risk. We also note that bone mineral density is not the only factor that impacts fracture risk; the architecture of the skeleton, alcohol usage, smoking, physical activity, and previous fractures are also major factors in fracture risk that are not represented in the model in this study [55].

### Clinical perspectives

Although estrogen replacement therapy (or hormone replacement therapy) can effectively manage menopause symptoms and post-menopausal osteoporosis, its benefits must be weighed against the heightened risks of blood clots, stroke, and breast cancer, especially with long-term use [56,57]. Furthermore, side effects such as weight gain, diarrhea, and mood changes can affect patient well-being and adherence [57]. The balance of risks and benefits associated with hormone replacement therapy varies considerably based on individual physiology, time since menopause onset, and the specific type of hormone replacement therapy [56]. Other current osteoporosis treatments, including antiresorptives and osteoanabolic therapies, have limitations on their duration of use, typically ranging from one to five years [58]. Therefore, the development of alternative long-term osteoporosis treatments are needed to broaden the range of effective options for patients. Our mathematical modeling analysis presented in this study supports clinical evidence suggesting that ARBs may contribute to maintaining bone health during post-menopausal estrogen decline by reducing AT1R-bound Ang II levels. An important future extension would be to investigate hormone replacement therapy, including the impact of treatment timing, dose, and administration route on the coupled RAS–calcium–bone remodeling system.

Indeed, there are a number of clinical applications that this model could be extended to study. The RAS is a key hormone system in blood pressure regulation. The RAS model used in this study is part of a larger model that involves whole-body blood pressure regulation [18,19]. Using a similar approach to [59], the model presented in this study may be incorporated into the blood pressure model [19] through the RAS to investigate the interactions of hypertension and bone remodeling. Chronic kidney disease (CKD) is a disorder of the loss of kidney function that affects over 10% of the global population [60]. Many patients with CKD experience CKD-metabolic bone disorder, where their impaired renal function results in loss of bone mineral density. Previous mathematical modeling studies have used the underlying calcium homeostasis and bone remodeling model to study this disorder [15,16,61]. For example, in [15], the authors simulated a progressive loss of renal function using an exponential decline in glomerular filtration rate (GFR). However, none of these studies have considered the RAS. Many CKD patients take RAS inhibitors to protect their kidney function, prevent hypertension, and have been shown to improve bone health [62]. Furthermore, CKD impacts the RAS, leading to decreased conversion of Ang I to Ang1–7 via neprilysin and increased conversion of Ang I to Ang II via ACE [63]. The model presented in this study may be used to investigate how RAS inhibitors may impact bone density and electrolyte balance in patients with CKD, by incorporating CKD mechanisms from [15,16,61] with additional CKD effects on the RAS [63].

### Limitations and future directions

In this study, we developed a mathematical model that describes the interactions between the RAS, calciotropic hormones, calcium regulation, and bone remodeling. One major limitation of this model is the large number of unknown parameters, resulting in uncertainty in the model outcomes. In this study, we analyzed the impacts of variations in parameter values on model output by conducting a global sensitivity analysis using the Sobol method. However, we do recognize that there may be parameters and mechanisms that are not included or could be changed in this model.

Several physiological quantities shown as fixed values in Fig 1 were treated as prescribed inputs or baseline parameters rather than dynamically regulated state variables. This modeling choice was made to limit model complexity and parameter uncertainty in this first integrated framework. Additionally, the present study focused on a representative post-menopausal estrogen decline trajectory rather than investigating inter-individual variability in menopause timing or decline rate. Future extensions could incorporate alternative estrogen decline regimes and dynamic regulation of additional physiological compartments.

In addition, while calcium and phosphate are dynamically regulated within the present framework, we did not investigate dedicated calcium or phosphate perturbation protocols, which could be explored in future studies to further characterize mineral homeostasis within the integrated model.

A worthwhile future direction is to accurately model an aging population. In this study, we conducted simulations that represent the estrogen decline that is seen in post-menopausal women. However, aging populations, including post-menopausal women, have shown other physiological changes related to the systems in the model presented in this study. For example, aging populations have shown a blunted response to ACE inhibitors, have a lower GFR, decreased intestinal calcium absorption, lower vitamin D levels, and higher PTH levels [64–66]. These effects are not included in the model developed in this study at this time. Indeed, future work may consider the specific physiological impacts of aging for a more comprehensive description of post-menopausal physiology.

Given the importance of estrogen and using a female-specific RAS, the model presented in this study only represents a female population. However, the same RAS subsystem could be used with the male-specific parameter set from [19]. Sex differences have been found in the RAS [67] and renal handling of calcium [68,69]. Men also can form osteoporosis as they age, which is not driven by the declining estrogen levels found in post-menopausal women. Rianon et al. [70] found that there are sex- and race-specific effects of ACEi on the prevention of age-related bone loss. Specifically, the authors reported that ACEi were effective at protecting against bone loss in African-American elderly men [70]. This is different than reports that did not consider sex and race differences and only reported significant changes in fracture risk in patients using ARB. Notably the systemic study by Wu et al. [12] found no significant difference in fracture risk in male patients on ACEi. The model presented in this study may be extended to consider how underlying sex and racial differences may explain altered treatment outcomes [71].

In this study, we do not include the hormone aldosterone, which is connected with the RAS as a part of the renin angiotensin aldosterone system. Aldosterone is released from the adrenal cortex in response to stimulation by AT1R-bound Ang II [72]. Researchers have shown that aldosterone also has a bidirectional relationship with the calciotropic hormones, as it both stimulates and is stimulated by PTH [6]. Additionally, aldosterone increases renal calcium reabsorption [73]. Clinical reports have shown that the drug spironolactone (an aldosterone inhibitor), has a significant impact on the bone health of hypertensive patients [74]. Indeed, this hormone may be added to this model to study the impacts of hypertension, spironolactone, hyperaldosteronism, or other relevant conditions and medications on bone density and calcium regulation. Additionally, in this study, we assume that the RAS activity in the bone is the same as the systemic RAS, though this may not always be the case. Experimental evidence has shown that there is a bone-specific RAS [75]. Future work may involve modeling a bone-specific RAS to consider the impacts on bone remodeling similar to the renal-specific RAS models in [17,76].

FGF-23 is produced by osteoblasts and osteocytes and has numerous system wide effects that impact the RAS, bone remodeling, and calcium homeostasis [77]. FGF-23 acts through the fibroblast growth factor receptor/αKlotho complex expressed in the kidneys and parathyroid glands. Effects include the suppression of calcitriol, PTH, and phosphate reabsorption. Furthermore, FGF-23 levels have been implicated as a clinically relevant biomarker for osteoporosis. High circulating FGF-23 levels are associated with an increased incidence of fragility fractures and decreased BMD [77]. Future model extensions could incorporate FGF-23 dynamics.

Additionally, the current model assumes that PTH directly stimulates renin secretion. However, experimental evidence has shown that PTH stimulates renin secretion through binding to juxtaglomerular PTH1R receptors [52]. Including explicit PTH1R-mediated signaling dynamics may better capture the complex endocrine feedback mechanisms governing calcium homeostasis and the renin-angiotensin system.

Estrogen levels vary during the different phases of the menstrual cycle in menstruating women [22]. Women who take oral contraceptives also have different estrogen levels [78]. The mathematical model presented here may be adapted to consider how changes in estrogen impacts the RAS, calcium homeostasis, and bone remodeling during the different phases of the menstrual cycle or on oral contraceptives [79]. During pregnancy, estrogen levels rise dramatically, while post-parturition, the estrogen levels drop below pre-pregnancy levels to support lactation [80]. Each of these changes results in major impacts on physiology, including the RAS, calcium homeostasis, and bone remodeling [80,81]. Previously, we developed mathematical models that represent calcium homeostasis in rats that consider sex differences and maternal adaptations of pregnancy and lactation [82], but these models do not include a detailed bone compartment or the impact of the RAS. The model presented in this study may be similarly adapted to consider the major changes that women undergo during pregnancy and lactation. Future work could further evaluate the model against longitudinal menopause-transition cohorts, such as SWAN, that include antihypertensive medication use and bone-related outcomes [83].

## Supporting information

S1 Appendix Model equations. (PDF)

S1 Table Key parameter descriptions. (PDF)

S2 Appendix Impact of declining estrogen levels on the RAS. (PDF)

S3 Appendix Additional figures. (PDF)
