# Supplementary material for: A physiology-based mathematical model of the renin-angiotensin system, bone remodeling, and calcium homeostasis: Effects of estrogen and renin-angiotensin system inhibitors
Source: PLoS Comput Biol. 2026 Sep 22;22(9):e1013101. doi: 10.1371/journal.pcbi.1013101 (PMC13623139; doi:10.1371/journal.pcbi.1013101)
Supplement: Supplementary file 1 [file pcbi.1013101.s001.pdf]

Model equations

A list of the ordinary differential equations in the model is given below.

A Renin-angiotensin system

$$\frac{d[\mathrm{PRC}]}{dt} = R_{\mathrm{sec}} - \frac{\ln(2)}{h_{\mathrm{renin}}} \cdot [\mathrm{PRC}] \tag{A.1}$$

where

$$R_{\mathrm{sec}} = N_{\mathrm{rs}} \cdot \nu_{\mathrm{AT1R}} \cdot g^{-}_{\mathrm{renin}}(E) \cdot H^{-}_{\mathrm{CaSR,renin}} \cdot H^{+}_{\mathrm{PTH,renin}} \cdot H^{-}_{\mathrm{Ctriol,renin}}. \tag{A.2}$$

.

$$\frac{d[\mathrm{AGT}]}{dt} = g^{+}_{\mathrm{AGT}}(E) \cdot k_{\mathrm{AGT}} - \mathrm{PRA} - \frac{\ln(2)}{h_{\mathrm{AGT}}} \cdot [\mathrm{AGT}] \tag{A.3}$$

where

$$\mathrm{PRA} = X_{\mathrm{PRCPRA}} \cdot [\mathrm{PRC}] \tag{A.4}$$

$$\frac{d[\mathrm{AngI}]}{dt} = \mathrm{PRA} - (g^{-}_{\mathrm{ACE}}(E) \cdot c_{\mathrm{ACE}} + c_{\mathrm{chym}} + c_{\mathrm{nep}})[\mathrm{AngI}] - \frac{\ln(2)}{h_{\mathrm{AngI}}}[\mathrm{AngI}] \tag{A.5}$$

$$\begin{aligned} \frac{d[\mathrm{AngII}]}{dt} &= (g^{-}_{\mathrm{ACE}}(E) \cdot c_{\mathrm{ACE}} + c_{\mathrm{chym}})[\mathrm{AngI}] \\ &- (c_{\mathrm{ACE2}} + c_{\mathrm{II,IV}} + g^{-}_{\mathrm{AT1}}(E) \cdot c_{\mathrm{AT1}} + g^{+}_{\mathrm{AT2}}(E) \cdot c_{\mathrm{AT2}})[\mathrm{AngII}] \\ &- \frac{\ln(2)}{h_{\mathrm{AngII}}}[\mathrm{AngII}] + k_{\mathrm{inf}} \end{aligned} \tag{A.6}$$

$$\frac{d[\mathrm{Ang}(1-7)]}{dt} = c_{\mathrm{NEP}} \cdot [\mathrm{AngI}] + c_{\mathrm{ACE2}} \cdot [\mathrm{AngII}] - \frac{\ln(2)}{h_{\mathrm{Ang}(1-7)}}[\mathrm{Ang}(1-7)] \tag{A.7}$$

$$\frac{d[\mathrm{AngIV}]}{dt} = c_{\mathrm{AngII=AngIV}} \cdot [\mathrm{AngII}] - \frac{\ln(2)}{h_{\mathrm{AngIV}}} \cdot [\mathrm{AngIV}] \tag{A.8}$$

$$\frac{d[\mathrm{AT1R-AngII}]}{dt} = g^{-}_{\mathrm{AT1}}(E) \cdot c_{\mathrm{AT1}}[\mathrm{AngII}] - \frac{\ln(2)}{h_{\mathrm{AT1}}}[\mathrm{AT1R-AngII}] \tag{A.9}$$

$$\frac{d[\mathrm{AT2R-AngII}]}{dt} = g^{+}_{\mathrm{AT2}}(E) \cdot c_{\mathrm{AT2}}[\mathrm{AngII}] - \frac{\ln(2)}{h_{\mathrm{AT2}}}[\mathrm{AT2R-AngII}] \tag{A.10}$$

B Calcium regulation

$$\frac{d[\mathrm{Ca}]}{dt} = \Phi_{\mathrm{Bone2Plas-Ca}} - \Phi_{\mathrm{Plas2Bone-Ca}} - \Phi_{\mathrm{Urine-Ca}} + \Phi_{\mathrm{Gut2Plas-Ca}} \tag{B.1}$$

with

$$\Phi_{\text{Gut2Plas-Ca}} = I_{\text{Ca}} \cdot \left(\Phi_{\text{Gut2Plas-Ca0}} + V_{\text{max-CtriolGut}} \cdot \frac{[\text{Ctriol}]^{\gamma_{\text{CtriolGut}}}}{(K_{\text{CtriolGut-Ca}})^{\gamma_{\text{CtriolGut}}} + [\text{Ctriol}]^{\gamma_{\text{CtriolGut}}}} \right) \tag{B.2}$$

$$\Phi_{\text{Plas2Bone-Ca}} = \xi_{15} \cdot [\text{Ca}] \cdot (1 - \nu_{\text{J15}} + \nu_{\text{J15}} \cdot \text{HAp}) \tag{B.3}$$

$$\Phi_{\text{Bone2Plas-Ca}} = \text{HAp} \cdot \left(\Phi_{\text{Bone2Plas-Ca0}} + V_{\text{max,OC-Ca}} \cdot \frac{\text{OC}^{\gamma_{\text{OCCa}}}}{K_{\text{OC}}^{\gamma_{\text{OCCa}}} + \text{OC}^{\gamma_{\text{OCCa}}}} \right) \tag{B.4}$$

$$\Phi_{\text{Urine-Ca}} = \kappa_{Ca} \cdot \Phi_{\text{GFR}} \cdot [\text{Ca}] - \Phi_{\text{Reabs-Ca}} \tag{B.5}$$

$$\tag{B.6}$$

where

$$\Phi^{\text{max}}_{\text{Reabs-Ca}} = \Phi_{\text{GFR}} \cdot [\text{Ca}] \cdot (1 + m_{\text{EST}}(E - 1)) \tag{B.7}$$

$$\Phi_{\text{Reabs-Ca}} = \Phi^{\text{max}}_{\text{Reabs-Ca}} \cdot \frac{V_{\text{max-Ca}} \cdot [\text{Ca}]}{K_{\text{reabs-Ca}} + [\text{Ca}]} \cdot \frac{V_{\text{max-PTHreabs}} \cdot [\text{PTH}]}{K_{\text{PTH-reabs}} + [\text{PTH}]} \tag{B.8}$$

C Parathyroid hormone

$$\frac{d[\text{PTH}]}{dt} = S_{\text{PTH}} \cdot H^{+}_{\text{AT1R,PTH}} \cdot H^{-}_{\text{CaSR,PTH}} - k_{\text{deg,PTH}} \cdot [\text{PTH}] \tag{C.1}$$

$$S_{\text{PTH}} = H^{-}_{\text{CaSR,PTH}} \cdot \frac{\text{PTG}}{0.5} \cdot \text{PT}_{\text{max}} \tag{C.2}$$

$$\frac{d\text{PTG}}{dt} = k_{\text{prod-PTG}} - k_{\text{deg-PTG}} \cdot \text{PTG} \tag{C.3}$$

$$\frac{d\text{PT}_{\text{max}}}{dt} = \text{PT}_{\text{max-base}} \cdot H^{-}_{\text{Ctriol,PTmax}} \cdot H^{+}_{\text{Phos,PTmax}} - k_{\text{deg-PTmax}} \cdot \text{PT}_{\text{max}} \tag{C.4}$$

D Calcitriol

$$\frac{d[\text{AOH}]}{dt} = k_{\text{prod-AOH}} - k_{\text{deg}} \cdot [\text{AOH}] \tag{D.1}$$

with

$$k_{\text{prod-AOH}} = k_{\text{base-AOH}} \cdot H^{+}_{\text{PTH,AOH}} \cdot H^{-}_{\text{Phos,AOH}} \tag{D.2}$$

$$\frac{d[\text{Ctriol}]}{dt} = \text{AOH} - k_{\text{deg-Ctriol}} \cdot [\text{Ctriol}] \tag{D.3}$$

E Phosphate regulation

$$\begin{aligned}\frac{d[\text{PhosECC}]}{dt} &= \Phi_{\text{Bone2Plas-Phos}} - \Phi_{\text{Plas2Bone-Phos}} - \Phi_{\text{Urine-Phos}} \\ &+ \Phi_{\text{Gut2Plas-Phos}} - \Phi_{\text{ECC2Int-Phos}} + \Phi_{\text{Int2ECC-Phos}}\end{aligned} \tag{E.1}$$

$$\frac{d[\text{PhosInt}]}{dt} = \Phi_{\text{ECC2Int-Phos}} - \Phi_{\text{Int2ECC-Phos}} \tag{E.2}$$

with

$$\Phi_{\mathrm{Gut2Plas-Phos}} = I_{\mathrm{Phos}} \cdot \left(\Phi_{\mathrm{Gut2Plas-Phos0}} + V_{\mathrm{max-CtriolGutPhos}} \cdot \frac{[\mathrm{Ctriol}]^{\gamma_{\mathrm{CtriolGut}}}}{(K_{\mathrm{CtriolGut-Ca}})^{\gamma_{\mathrm{CtriolGut}}} + [\mathrm{Ctriol}]^{\gamma_{\mathrm{CtriolGut}}}} \right) \tag{E.3}$$

$$\Phi_{\mathrm{Urine-Phos}} = \kappa_{\mathrm{phos}} \cdot \Phi_{\mathrm{GFR}} \cdot [\mathrm{ECCPhos}] - \Phi_{\mathrm{Phos-reabs}} \tag{E.4}$$

$$\Phi_{\mathrm{Plas2Bone-Phos}} = \eta_{\mathrm{bone-phos}} \cdot \Phi_{\mathrm{Plas2Bone-Ca}} \tag{E.5}$$

$$\Phi_{\mathrm{Bone2Plas-Phos}} = \eta_{\mathrm{bone-phos}} \cdot \Phi_{\mathrm{Bone2Plas-Ca}} \tag{E.6}$$

where

$$\Phi_{\mathrm{Phos-reabs}} = \Phi_{\mathrm{Phos-reabs0}} \cdot \Phi_{\mathrm{GFR}} \tag{E.7}$$

F Bone remodeling

$$
\begin{aligned}
\frac{d\mathrm{OC}}{dt} &= k_{\mathrm{prod-OC}} \cdot H^{+}_{\mathrm{RANK-RANKL}} \cdot \mathrm{OC}_{\mathrm{base}} \\
&\quad - k_{\mathrm{deg-OC}} \cdot H^{+}_{\mathrm{TGFBact,OC}} \cdot H^{-}_{\mathrm{RNK-RNKL}} \cdot \mathrm{OC} && \text{(F.1)}
\end{aligned}
$$

$$
\frac{d\mathrm{pOB}}{dt} = k_{\mathrm{prod-pOB}} \cdot \left(\frac{1}{E}\right)^{\gamma_{\mathrm{pOB,EST}}} - k_{\mathrm{deg,pOB}} \cdot H^{+}_{\mathrm{TGFBact,pOB}} \cdot \mathrm{pOB} \qquad \text{(F.2)}
$$

$$
\frac{d\mathrm{OB}}{dt} = k_{\mathrm{prod-OB}} \cdot H^{-}_{\mathrm{TGFBact,OB}} \cdot \mathrm{pOB} - k_{\mathrm{deg-OB}} \cdot \mathrm{OB} \qquad \text{(F.3)}
$$

$$
\begin{aligned}
\frac{d\mathrm{RNK}}{dt} &= -k_3 \cdot \mathrm{RNK} \cdot \mathrm{L} + k_4 \cdot \mathrm{RNK-RNKL} \\
&\quad + k_{\mathrm{prod-RNK}} \cdot H^{+}_{\mathrm{TBFBact,RNK}} - k_{\mathrm{deg-RNK}} \cdot \mathrm{RNK} && \text{(F.4)}
\end{aligned}
$$

$$
\begin{aligned}
\frac{d\mathrm{RNKL}}{dt} &= -k_3 \cdot \mathrm{RNK} \cdot \mathrm{L} + k_4 \cdot \mathrm{RNK-RNKL} && \text{(F.5)} \\
&\quad + k_{\mathrm{prod-L}} \cdot \left(\frac{\mathrm{OB}}{\mathrm{OB}_0}\right)^{\gamma_{\mathrm{OBeff}}} \cdot H^{+}_{\mathrm{PTH,L}} \cdot H^{+}_{\mathrm{AT1R,RANKL}} \\
&\quad - k_{\mathrm{deg-L}} \cdot \mathrm{RNKL} - k_1 \cdot \mathrm{OPG} \cdot \mathrm{L} + k_2 \cdot \mathrm{RNK-OPG} && \text{(F.6)}
\end{aligned}
$$

$$
\frac{d\mathrm{RNK-RNKL}}{dt} = k_3 \cdot \mathrm{RNK} \cdot \mathrm{L} - k_4 \cdot \mathrm{RNK-RNKL} \qquad \text{(F.7)}
$$

$$
\begin{aligned}
\frac{d\mathrm{OPG}}{dt} &= -k_1 \cdot \mathrm{OPG} \cdot \mathrm{RNKL} + k_2 \cdot \mathrm{RNK-OPG} + \\
&\quad k_{\mathrm{prod-OPG}} \cdot H^{+}_{\mathrm{PTH,OPG}} \cdot H^{+}_{\mathrm{AT1R,OPG}} - k_{\mathrm{deg-OPG}} \cdot \mathrm{OPG} && \text{(F.8)}
\end{aligned}
$$

$$
\frac{d\mathrm{RNK-OPG}}{dt} = k_1 \cdot \mathrm{OPG} \cdot RNKL - k_2 \cdot \mathrm{RNK-OPG} \qquad \text{(F.9)}
$$

$$
\begin{aligned}
\frac{d\mathrm{TGFB}}{dt} &= k_{\mathrm{prod-TGF}} \cdot \left(\frac{\mathrm{OB}}{\mathrm{OB}_{\mathrm{base}}}\right)^{\gamma_{\mathrm{OBtgf}}} \cdot \left(\frac{1}{E}\right)^{\gamma_{\mathrm{TGFB,E}}} && \text{(F.10)} \\
&\quad - k_{\mathrm{act,TGFB}} \cdot \mathrm{TGFB} \cdot \left(\frac{\mathrm{OC}}{\mathrm{OC}_{\mathrm{base}}}\right)^{\gamma_{\mathrm{OCtgf}}} \cdot E^{\gamma_{\mathrm{tgfbactE}}} && \text{(F.11)}
\end{aligned}
$$

$$
\begin{aligned}
\frac{d\mathrm{TGFB}_{\mathrm{active}}}{dt} &= k_{\mathrm{act,TGFB}} \cdot \mathrm{TGFB} \cdot \left(\frac{\mathrm{OC}}{\mathrm{OC}_{\mathrm{base}}}\right)^{\gamma_{\mathrm{OCtgf}}} \cdot E^{\gamma_{\mathrm{tgfbactE}}} \\
&\quad - k_{\mathrm{deg-TGFact}} \cdot \mathrm{TGFB}_{\mathrm{active}} && \text{(F.12)}
\end{aligned}
$$

$$
\frac{d\mathrm{BMD}}{dt} = k_{\mathrm{BMD-form}} \cdot \left(\frac{\mathrm{OB}}{\mathrm{OB}_{\mathrm{base}}}\right)^{\gamma_{\mathrm{OBform}}} - k_{\mathrm{BMD-res}} \cdot \left(\frac{\mathrm{OC}}{\mathrm{OC}_{\mathrm{base}}}\right)^{\gamma_{\mathrm{OBres}}} \cdot \mathrm{BMD} \qquad \text{(F.13)}
$$

G List of variable names

- AGT: angiotensinogen
- Ang I: angiotensin I
- Ang II: angiotensin II
- Ang (1-7): angiotensin (1-7)
- Ang IV: angiotensin IV
- AOH: 1α-hydroxylase
- AT1R-AngII: angiotensin type 1 receptor bound Ang II
- AT2R-AngII: angiotensin type 2 receptor bound Ang II
- BMD: bone mineral density
- Ca: plasma calcium
- Ctriol: calcitriol in plasma
- E: normalized estrogen level
- OB: osteoblasts
- OC: osteoclasts
- OPG: osteoprotegerin
- PhosECC: extracellular phosphate
- PhosInt: intracellular phosphate
- pOB: pre-osteoblasts
- PRA: plasma renin activity
- PRC: plasma renin concentration
- PT_{max}: maximal parathyroid gland secretion
- PTG: parathyroid hormone in the parathyroid glands
- PTH: parathyroid hormone
- RNK: Receptor activator of nuclear factor kappa-B
- RNKL: Receptor activator of nuclear factor kappa-B ligand
- TGFB: latent transforming growth factor β
- $\text{TGFB}_{\text{active}}$: active transforming growth factor β
