# Supplementary material for: A physiology-based mathematical model of the renin-angiotensin system, bone remodeling, and calcium homeostasis: Effects of estrogen and renin-angiotensin system inhibitors
Source: PLoS Comput Biol. 2026 Sep 22;22(9):e1013101. doi: 10.1371/journal.pcbi.1013101 (PMC13623139; doi:10.1371/journal.pcbi.1013101)
Supplement: Supplementary file 2 [file pcbi.1013101.s002.pdf]

Key parameter descriptions

Name	Description	Reference
$E_{\max,\mathrm{L-PTH}}$	half-maximal response of effect of PTH on RANKL production	[13]
k_3	binding rate of RANK and RANKL	[13]
k_4	unbinding rate of RANK-RANKL	[13]
$k_{\mathrm{in,L}}$	baseline RANKL production	[13]
$k_{\mathrm{out,L}}$	degradation rate of RANKL	[13]
$k_{\mathrm{in,RNK}}$	baseline RANK production	[13]
$k_{\mathrm{out,RNK}}$	degradation rate of RANK	[13]
$\rho_{\mathrm{AT1R,L}}$	minimum impact of AT1R on RANKL	Eq. 23
$\delta_{\mathrm{AT1R,L}}$	half-maximal response of AT1R impact on RANKL	Eq. 23
$\alpha_{\mathrm{CaSR,renin}}$	maximum impact of CaSR on renin	Eq. 19
$\delta_{\mathrm{CaSR,renin}}$	half-maximal response of CaSR impact on renin	Eq. 19
$\Phi_{\mathrm{Plas2BoneCa0}}$	baseline calcium exchange from extracellular fluid to bone	[13]
$\Phi_{\mathrm{Bone2PlasCa0}}$	baseline calcium exchange from bone to extracellular fluid	[13]
c_{AT1R}	binding rate of Ang II to AT1R	[18,19], Eq. 10
N_{rs}	baseline renin secretion	[18,19], Eq. 4
X_{PRCPRA}	equilibrium ratio of PRA to PRA	[18]
$\delta_{\mathrm{PTH,renin}}$	half-maximal response of PTH impact on renin	Eq. 20
$K_{\mathrm{Ca,PTH}}$	half-maximal response of PTH secretion to CaSR	[13]
$k_{\mathrm{base,AOH}}$	baseline AOH production	[13]
$k_{\mathrm{deg,AOH}}$	degradation rate of AOH	[13]
$V_{\max,\mathrm{PTH-AOH}}$	maximum effect of PTH on AOH production	[13]
$\delta_{\mathrm{AOH,Phos}}$	half-maximal response of impact of phosphate on AOH	[13]
$V_{\max,\mathrm{Phos}}$	maximum impact of phosphate on AOH production	[13]
$V_{\max,\mathrm{PTH-reabs}}$	maximum impact of PTH on renal reabsorption	[13]

Table : Descriptions of parameters shown in S4 Figs. C, D, and E. The reference is the original source of the parameter value. Ang II: angiotensin II, AOH: 1α-hydroxylase, AT1R: angiotensin type 1 receptor, CaSR: calcium sensing receptor, OC: osteoclast, PTH: parathyroid hormone
