# Supplementary material for: A physiology-based mathematical model of the renin-angiotensin system, bone remodeling, and calcium homeostasis: Effects of estrogen and renin-angiotensin system inhibitors
Source: PLoS Comput Biol. 2026 Sep 22;22(9):e1013101. doi: 10.1371/journal.pcbi.1013101 (PMC13623139; doi:10.1371/journal.pcbi.1013101)
Supplement: Supplementary file 3 [file pcbi.1013101.s003.pdf]

Impact of declining estrogen levels on the RAS

How do the various impacts of estrogen on the RAS change RAS levels during post-menopausal estrogen decline? To investigate this question, we conducted a simulations of the estrogen-RAS model presented in this study. The results are shown in Fig. A. We assume that estrogen decline starts at age 50 years (Fig. A(A); Eq. 1). The decline in estrogen levels, decreases AGT production so that by age 80 years [AGT] is 60% below pre-menopausal levels (Fig. A(B)). Plasma renin secretion is inhibited by estrogen levels, which are rapidly decreasing. Renin secretion is inhibited by estrogen, so the lowered estrogen levels result in driving increased renin secretion so that ultimately plasma renin levels rise (Fig. A(C)).

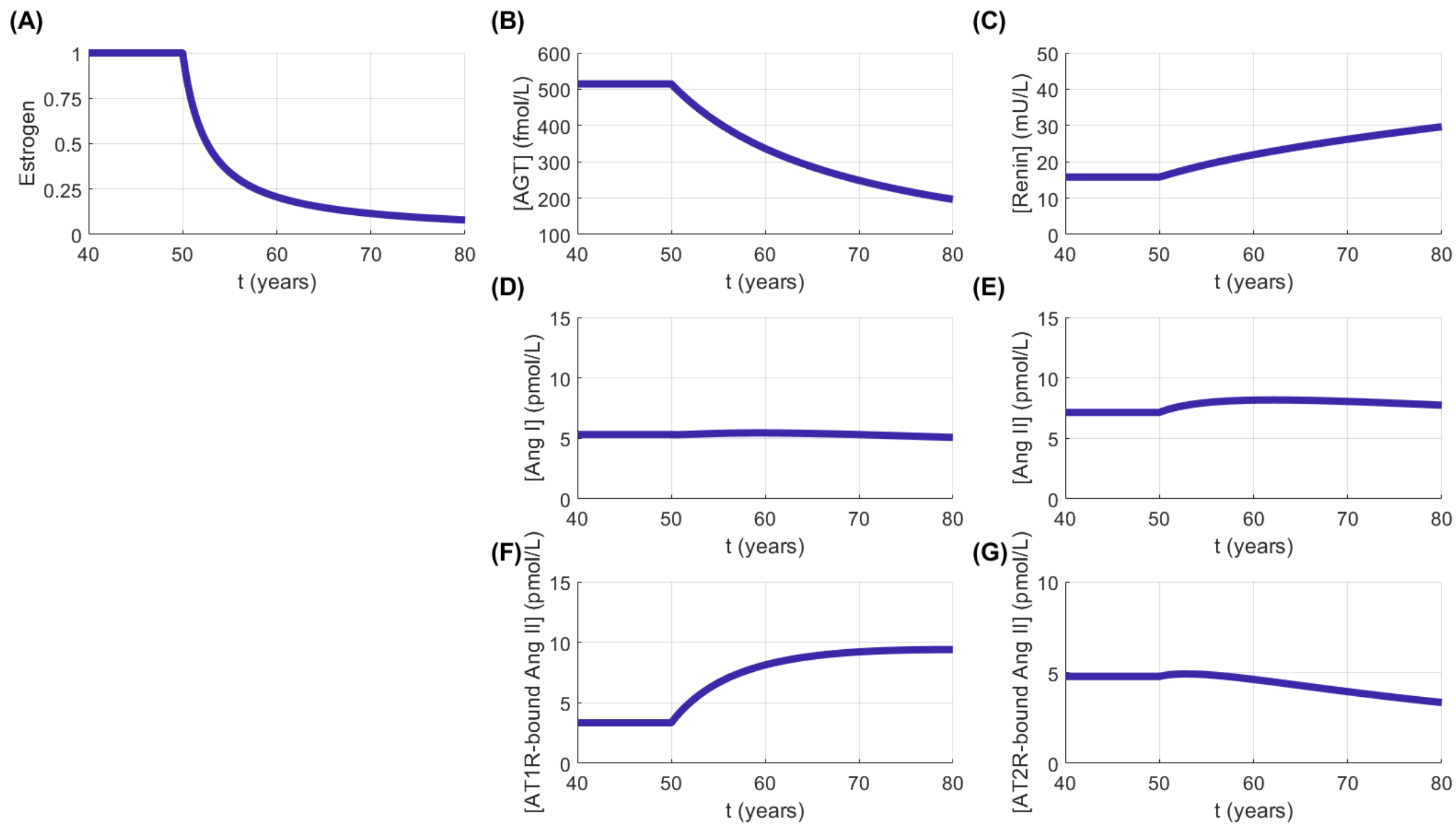

Fig A: Simulation results showing the impact of decreasing (A) estrogen levels on the plasma concentration of (B) AGT, (C) renin, (D) Ang I, (E) Ang II, (F) AT1R-bound Ang II, and (G) AT2R-bound Ang II. AGT: angiotensinogen, Ang: angiotensin, AT1R: angiotensin type 1 receptor, AT2R: angiotensin type 2 receptor

While renin concentration increases, the decreased AGT levels result in decreased PRA, which drives Ang I production. This results in stable Ang I levels (Fig. A(D)). ACE activity is increased so we see a slight increase in Ang II levels (Fig. A(E)). Ang II then binds to AT1R and AT2R. Estrogen inhibits AT1R binding and activates AT2R binding (Fig. 1). When estrogen levels decrease, AT1R receptors are inhibited less, thus leading to increased AT1R-bound Ang II levels (Fig AF). For AT2R receptors, initially, AT2R-bound Ang II levels slightly increase from baseline due to the increased Ang II levels, but later AT2R-bound Ang II levels decrease from baseline due to the loss of activation by estrogen levels (Fig. A(G)).

We conducted a Sobol sensitivity analysis on the estrogen-RAS model to investigate which parameters impact the estrogen deficiency model simulation the most for AGT, Ang II, AT1R-bound Ang II, and AT2R-bound Ang II. We varied the parameters that represent the rate of enzyme impacts on the RAS (i.e., parameters c_i) as well as the parameters that represent estrogen

impact on the RAS (i.e., parameters e_i listed in Table 1). Each parameter was varied by a 2-fold increase and decrease from the baseline value. We then conducted a Sobol analysis at the time point of every 10 years for the estrogen deficiency simulation.

The results for the total order Sobol indices (S_{total}) over the estrogen decline RAS simulation are shown in Fig. B. Before the onset of estrogen decline due to menopause (i.e., $t < 50$), the parameters e_i (i= renin, AGT, ACE, etc), which represent the impact of estrogen on the RAS (Eqs. 2–3), do not have any impact on the model variation as expected because $E = 1$.

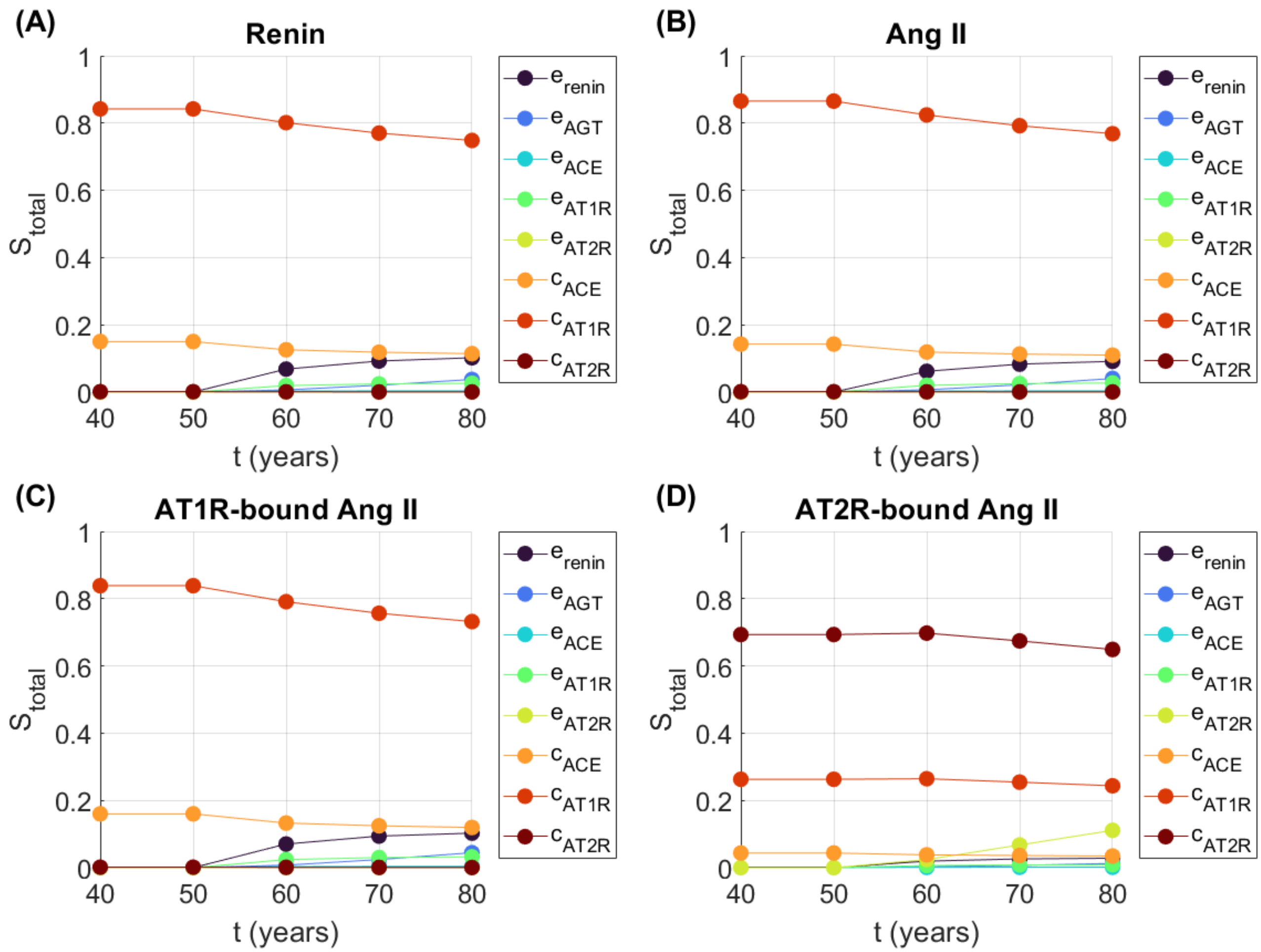

Fig B: Sobol total indices (S_{total}) for estrogen impact on (A) AGT, (B) Ang II, (C) AT1R-bound Ang II, and (D) AT2R-bound Ang II at different time points in the simulation. Parameters not shown did not have a S_{total} over 0.01 for any variable. AGT: angiotensinogen, Ang: angiotensin, AT1R, angiotensin type 1 receptor, AT2R: angiotensin type 2 receptor

For renin, e_{renin} represents the impact of estrogen on renin secretion and leads to about 10% of the model output variation by $t = 70$ years (Fig. B(A)). The parameters with the largest output on model output of Ang II are c_{ACE} and c_{AT1R} which correspond to ACE activity and AT1R binding, respectively (Fig. B(B)). After the decline in estrogen, the impact of estrogen on renin secretion, e_{renin} contributes to the variation in the model output of Ang II as well (Fig. B(B)). AT1R-bound Ang II is also impacted by c_{ACE} and c_{AT1R} the most due to the dependence on Ang II levels (Fig. B(C)). After the onset of estrogen decline, the impact of estrogen on renin and AT1R binding contribute to variation in the model output. By the end of the simulation time, about

10.2% and 3.2% of model output variation of AT1R-bound Ang II can be attributed to e_{renin} and e_{AT1R}, respectively (Fig. B(C)). For AT2R-bound Ang II, about 11% of the total variation can be attributed to the impact of estrogen on AT2R binding, e_{AT2R}, by the end of the simulation time (Fig. B(D)).
