# Supplementary material for: A physiology-based mathematical model of the renin-angiotensin system, bone remodeling, and calcium homeostasis: Effects of estrogen and renin-angiotensin system inhibitors
Source: PLoS Comput Biol. 2026 Sep 22;22(9):e1013101. doi: 10.1371/journal.pcbi.1013101 (PMC13623139; doi:10.1371/journal.pcbi.1013101)
Supplement: Supplementary file 4 [file pcbi.1013101.s004.pdf]

Additional Figures

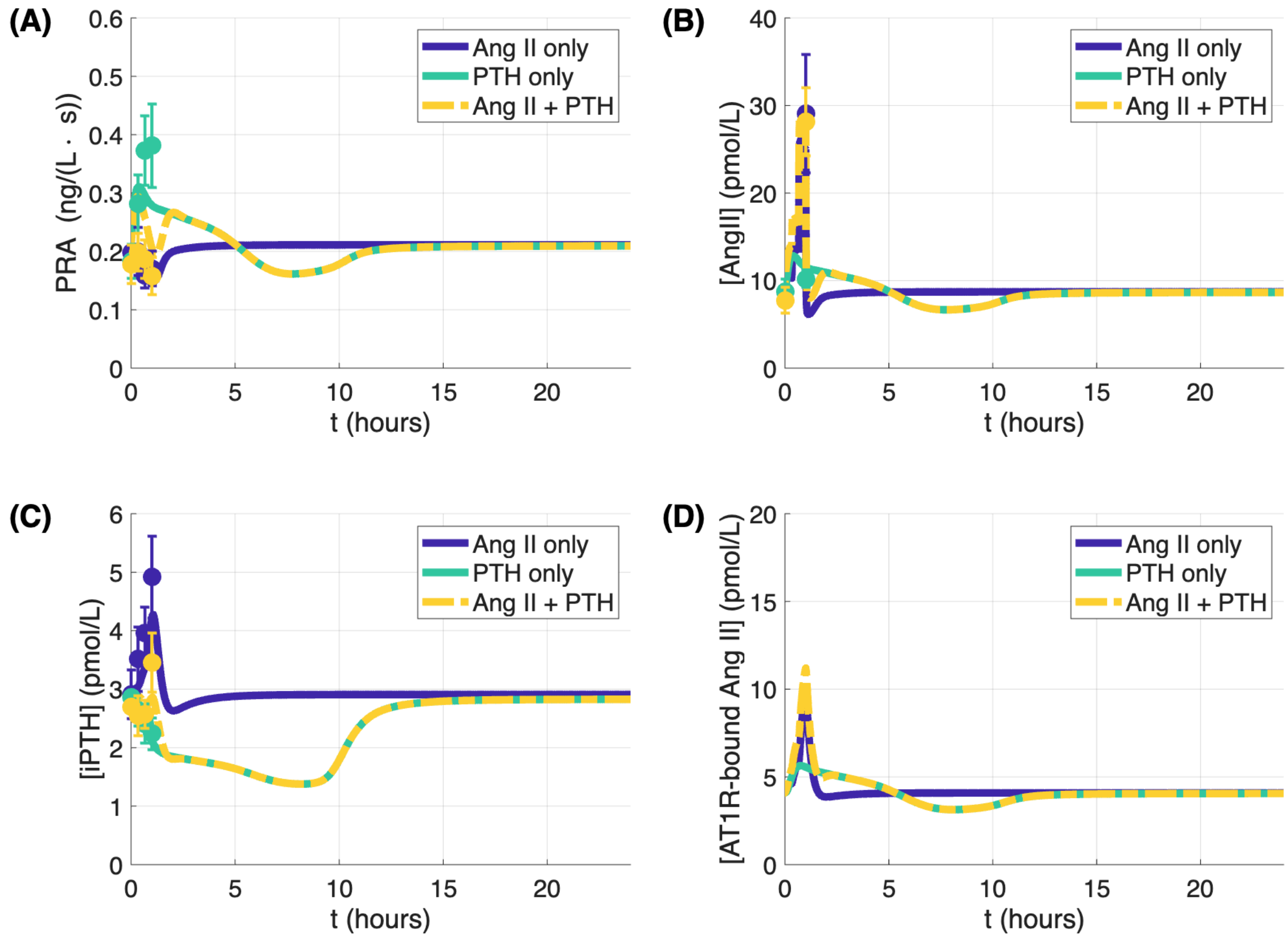

Fig A: Replication of Fig. 2 for a longer simulation time. Simulation results for replication of the infusion experiments in Grant et al. [40]. Results for (A) plasma renin activity (PRA), (B) angiotensin II (Ang II) plasma concentration, (C), parathyroid hormone (PTH) concentration, and (D) angiotensin receptor type II bound Ang II are shown during and following 1 hour infusions of Ang II only, PTH only, and combined Ang II and PTH infusions . Lines indicate simulations, while the dots and error bars in panels A–C are from the relevant experimental data presented in Grant et al. [40]. AT1R: angiotensin receptor type 1, iPTH: intact parathyroid hormone

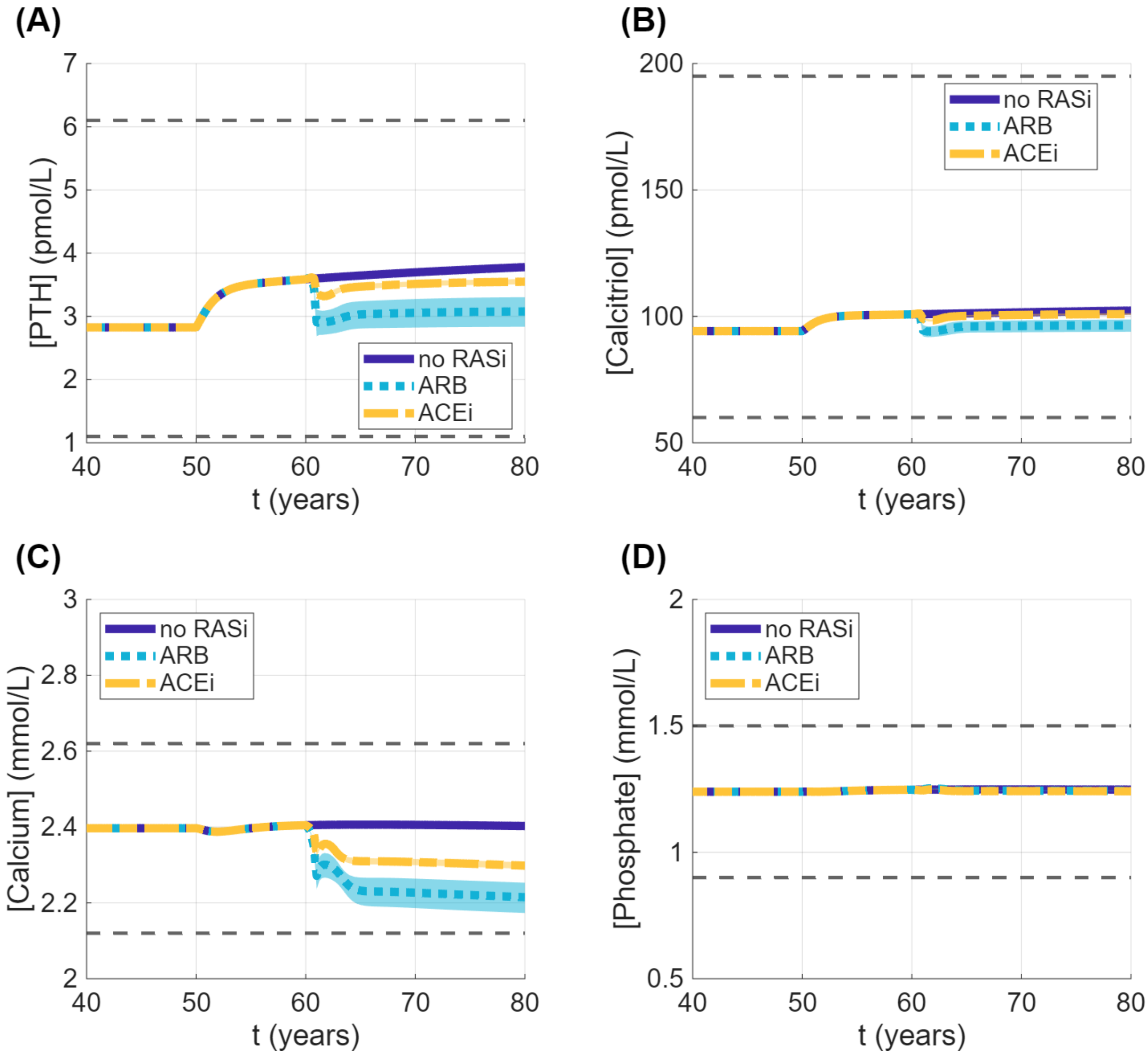

Fig B: Impact of estrogen deficiency and RAS inhibitors on extracellular levels of calciotropic hormones (A) parathyroid hormone and (B) calcitriol as well as electrolytes (C) calcium and (D) phosphate. For the angiotensin receptor blocker simulations (labeled "ARB" in the legend), σ_{ARB} *was varied between 92–98.3% based on the ranges reported in [18]. For the angiotensin-converting enzyme inhibitor simulations (labeled "ACEi" in the legend),* σ_{ACEi} *was varied between 94–97.2% as reported in [18]. Lines represent the mean values over the varied* σ_i *values for the respective treatment with shaded area representing the range. PTH: parathyroid hormone, RASi: RAS inhibitor, ARB: angiotensin receptor blocker, ACEi: angiotensin-converting enzyme inhibitor*

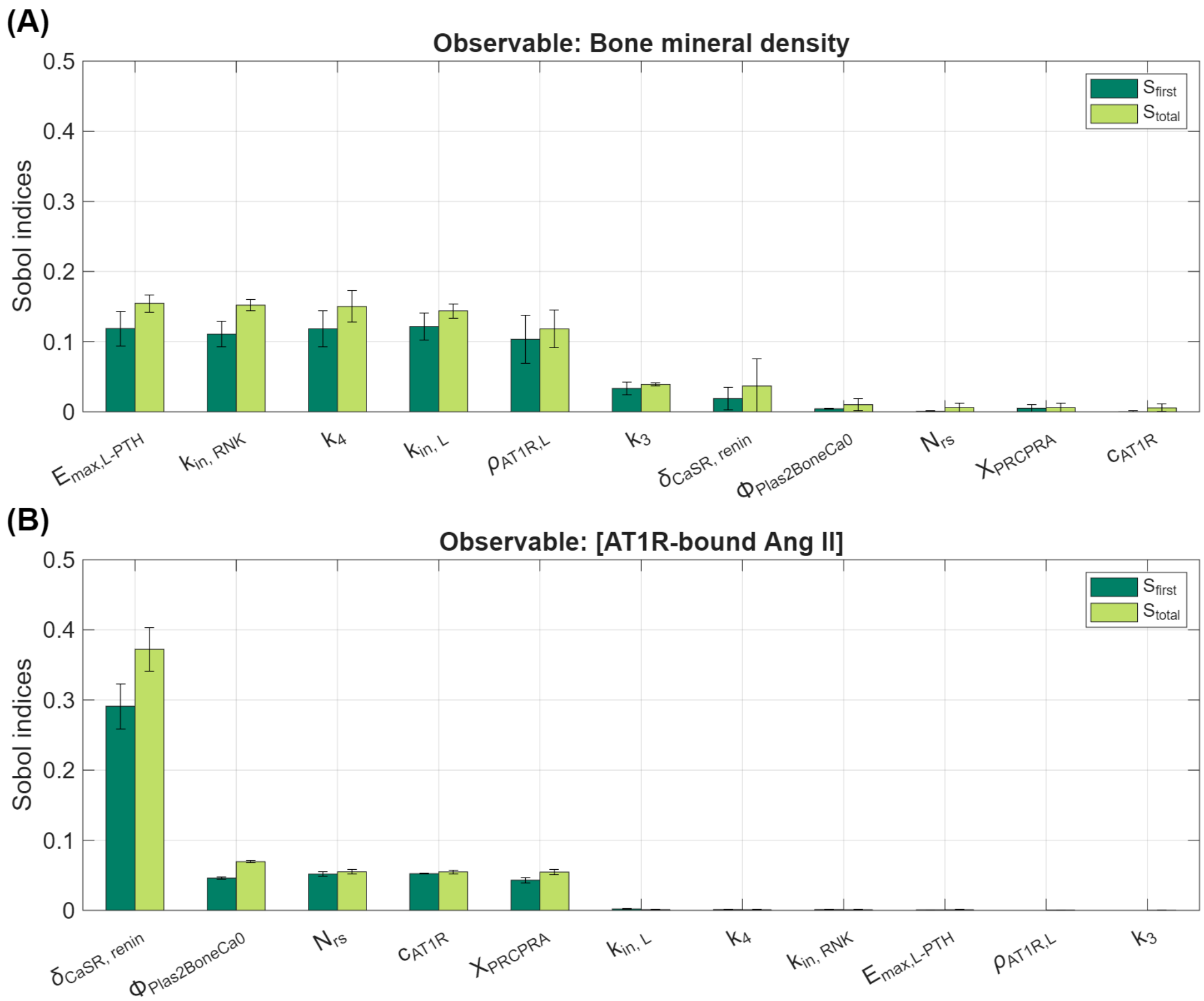

Fig C: Mean (bar) and standard deviation (error bar) of first and total order Sobol indices (denoted by S_{first} and S_{total} in legend, respectively) of the full model ran from age 20 to 80 years with estrogen decline. Sobol indices are shown for output variables (A) femoral neck bone mineral density and (B) AT1R-bound Ang II. Parameters shown had a maximal value of $S_{\text{total}} > 0.05$ for at least one output variable. A description of the parameters is given in Table . AT1R: angiotensin type 1 receptor, Ang II: angiotensin II

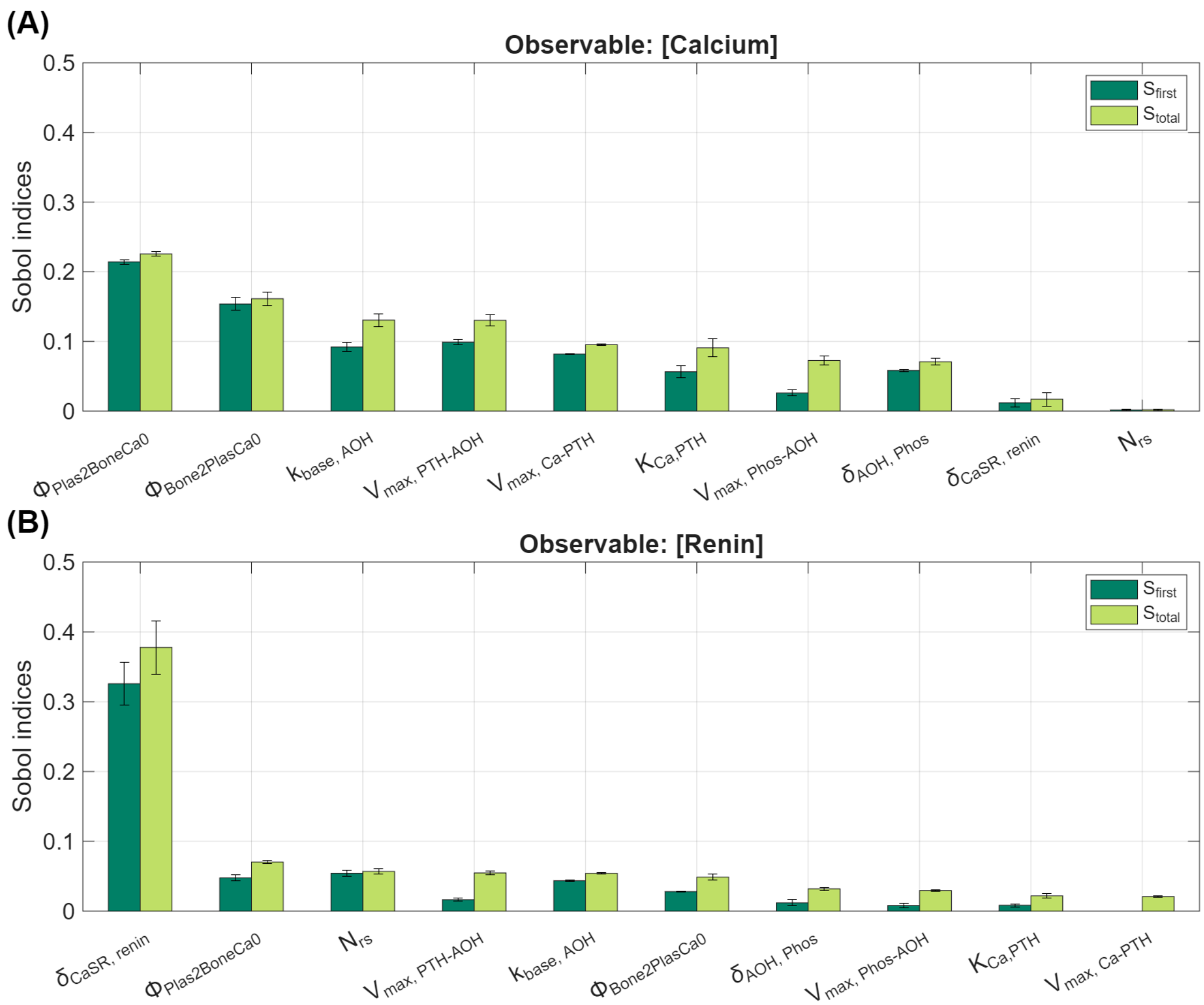

Fig D: Mean (bar) and standard deviation (error bar) of first and total order Sobol indices (denoted by S_{first} and S_{total} in legend, respectively) of full model simulation from age 20 to 80 years with estrogen decline. Parameters shown had a maximal value of $S_{\text{total}} > 0.05$ for at least one output variable. A description of the parameters is given in Table .

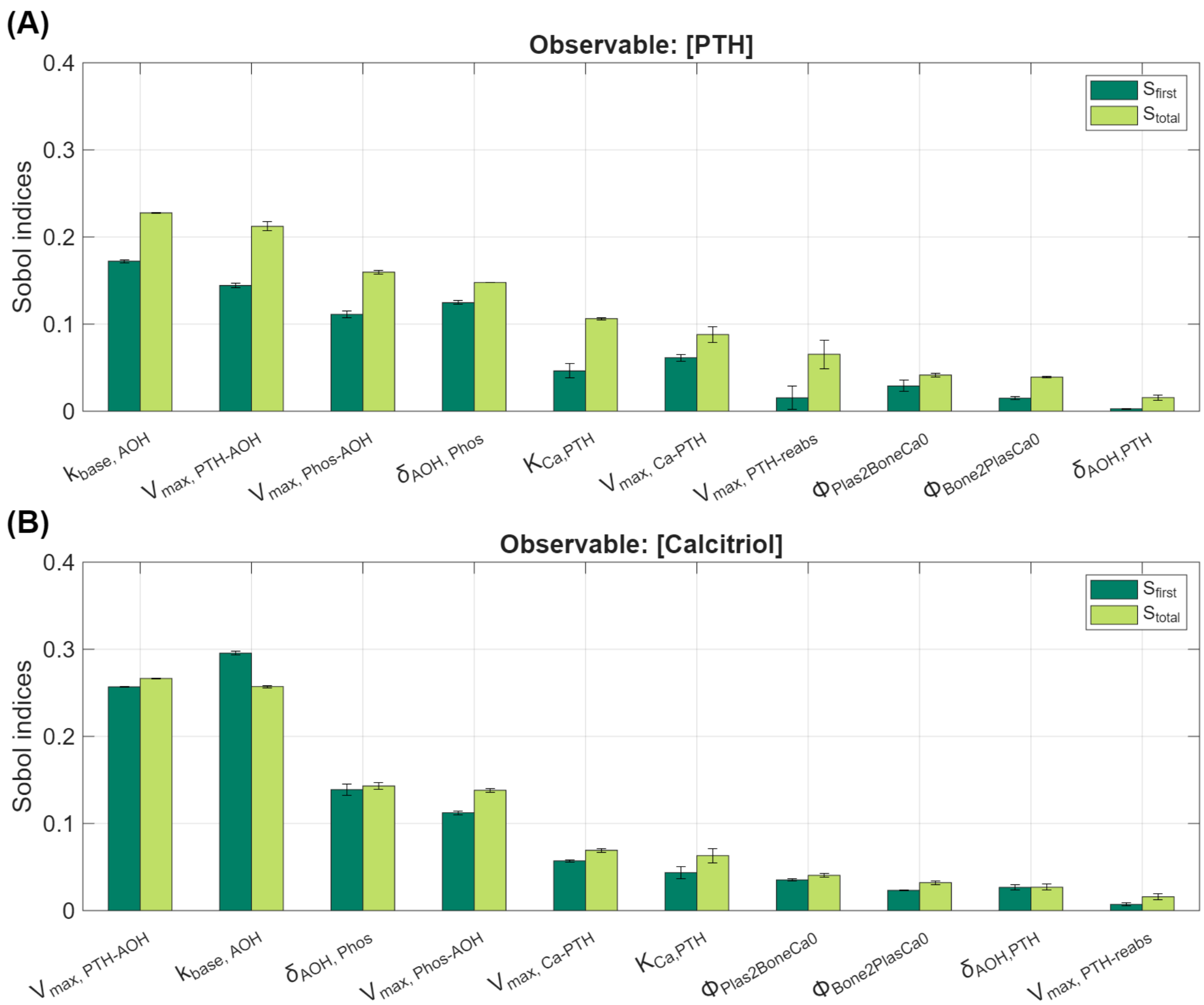

Fig E: Mean (bar) and standard deviation (error bar) of first and total order Sobol indices (denoted by S_{first} and S_{total} in legend, respectively) of the full model ran from age 20 to 80 years with estrogen decline. Sobol indices are shown for output variables (A) parathyroid hormone and (B) calcitriol. Parameters shown had a maximal value of $S_{\text{total}} > 0.05$ for at least one output variable. A description of the parameters is given in Table . PTH: parathyroid hormone
